# Supplementary material for: 2P-FENDO-II: A fiber bundle microscope for all-optical, large field-of-view brain studies in freely moving mice
Source: Cell Rep Methods. 2026 Feb 13;6(2):101305. doi: 10.1016/j.crmeth.2026.101305 (PMC12946751; doi:10.1016/j.crmeth.2026.101305)
Supplement: Document S1. Figures S1–S4 [file mmc1.pdf]

Cell Reports Methods, Volume *6*

Supplemental information

2P-FENDO-II: A fiber bundle microscope for all-optical, large field-of-view brain studies in freely moving mice

François G.C. Blot, Dimitri Decombe, Antonio Lorca-Cámara, Maya Anquetil, Vincent de Sars, Christophe Tourain, Benoît C. Forget, Nicolò Accanto, and Valentina Emiliani

SUPPLEMENTAL INFORMATION

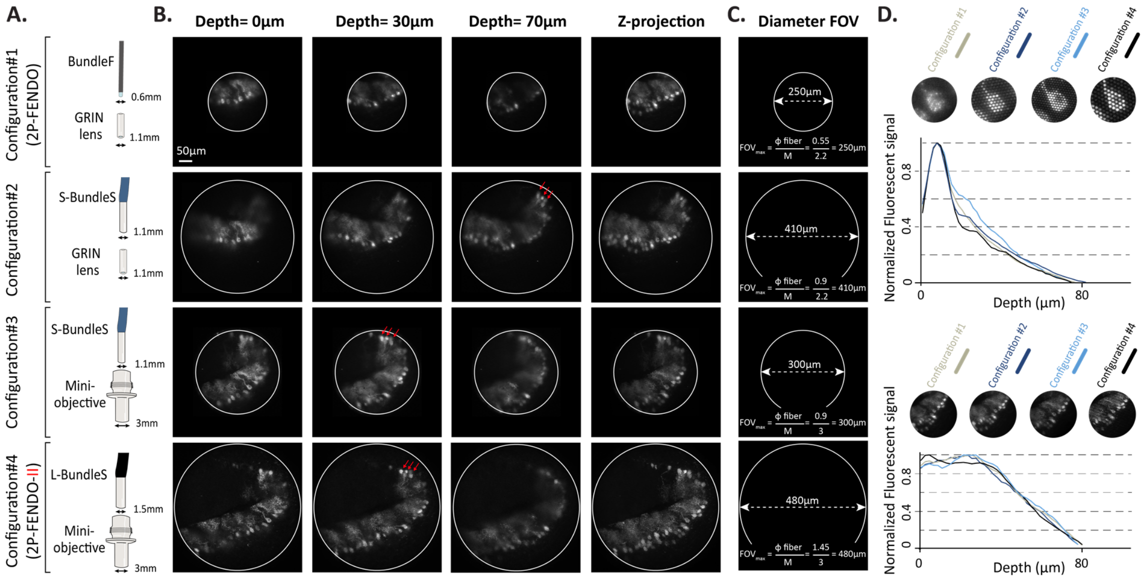

Figure S1: Imaging capability comparison between multiple configurations of the 2P-FENDO, related to Table 2.

(A). Schematic representation of the fibers and lenses for configuration#1 (BunfleF + GRIN lens), configuration#2 (S-BundleS + GRIN lens), configuration#3 (S-BundleS + min-objective), and configuration#4 (L-BundleS + mini-objective), with the corresponding external diameter value.

(B). Imaging on the same GCaMP6-positive 100 µm cerebellar slice with all proposed configurations of the system at multiple depth from the section's surface (0, 30 and 70µm), with z-projection. Red arrows point group of cells in periphery of the FOVs focus at 70µm for configuration#2, and 30µm for configurations #3 and #4, to illustrate the field curvature effect of the GRIN lens.

(C). Calculated diameter of each FOV. Configuration#4 is kept for all subsequent experiment and named 2P-FENDO-II.

(D). Normalized fluorescence signal relative to the depth of imaging tested independently for each configuration on the cerebellar slices of 80 µm. Signals were taken for a small ROI over a single Purkinje Cell (upper), or as an average of a 100µm ROI centred in the FOV (lower).

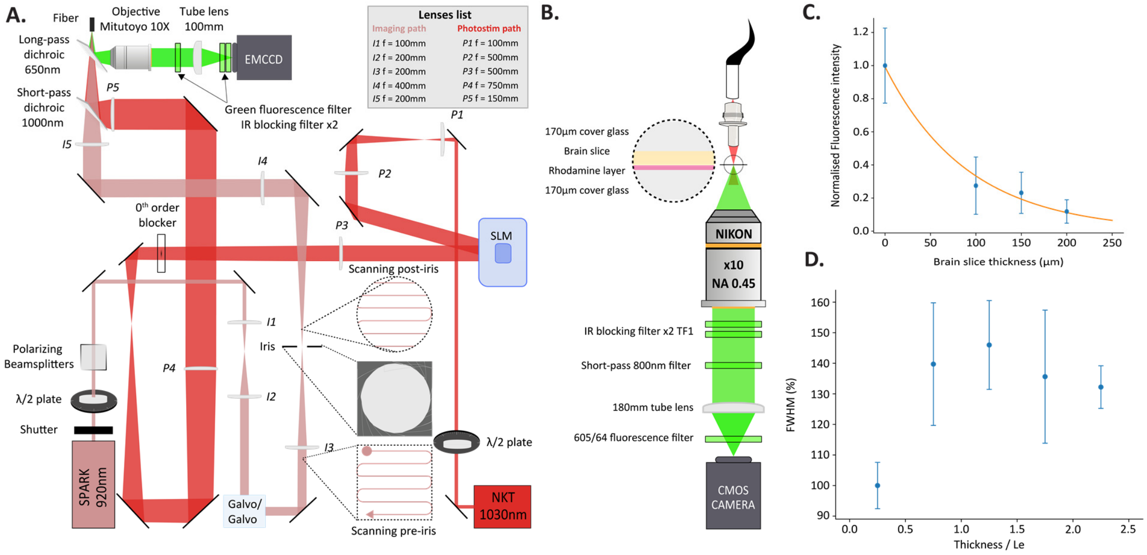

Figure S2: Description of the setup and the evolution of axial resolution with scattering tissue, related to Figure 1 and STAR★METHODS setup description.

(A). Complete schematic of the 2P-FENDO-II setup.

(B). Schematic of the transmissive configuration used to measure the evolution of the axial confinement of 2P photostimulation spot as a function of the brain slices thickness.

(C). Evolution of the fluorescence intensity signal with increasing brain slice thickness. Intensity values were averaged over multiple spot positions within the same FOV and across multiple FOVs. The orange curve represents an exponential decay fit corresponding to an attenuation length of Le=184µm.

(D). Broadening of the axial resolution as a function of penetration depth. To account for variations in scattering length among different preparations, the x-axis shows the brain slice thickness depth normalized to the corresponding attenuation length (Le). The y-axis represents the percentage increase in axial FWHM relative to the value in the absence of scattering. Based on this data and previously reported attenuation lengths for in vivo brain tissue (typically ~200 µm at 920 nm; Horton et al.[1]), we estimate that at the working depth of 120 µm used in cortical experiments, the axial broadening remains around ~35%.

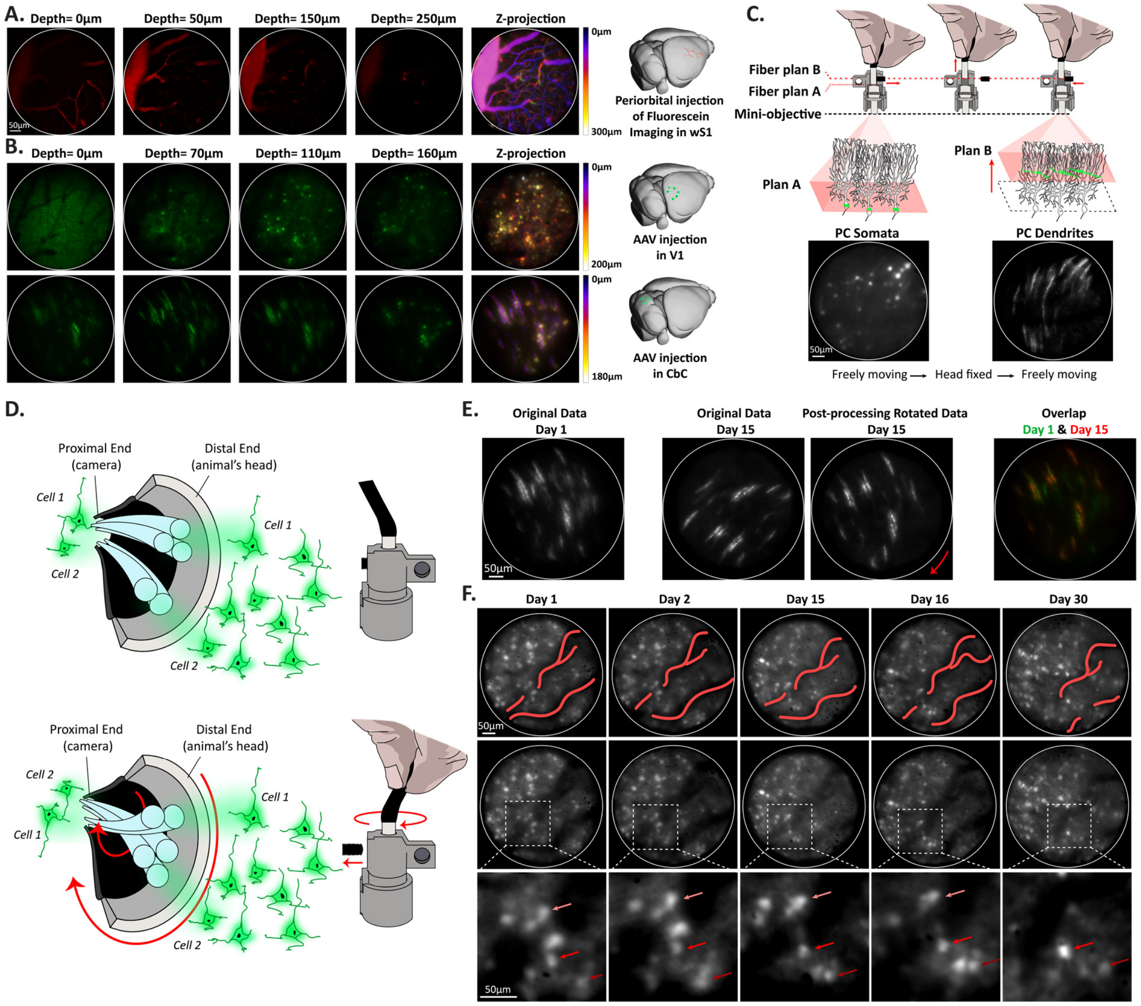

Figure S3: Imaging limits and FOV retrieval technique description, related to Figure 2 and 3.

(A). Z-stack imaging of head fixed mice at 130mW imaging scanning power, periorbitally injected with fluorescein to label blood vessels, with a cranial window over wS1. The images show multiple depths from the surface, and a z-projection of the whole stack color-coded for depth.

(B). Images of jGCaMP7s-labelled neurons in the V1 cortex image at 130mW scanning power (top row) or CbC at 65mW scanning power (bottom row) at multiple depths from the surface, with a z-projection of the whole stack color-coded for depth. Schematic representation of the imaging FOV location for blood vessels or neurons imaging are showed on the right.

(C). Demonstration of the system's flexibility in adjusting the imaging plane depth, with projections of acquisitions at plane A (estimated at 160µm from the surface) and plane B (estimated 50µm<x<90µm from the surface, see also Movie 2).

(D). Schematic illustrating the potential rotational effect of the FOV following manual rotation of the fiber.

(E). Acquisitions of the same FOV and CbC preparation taken 15 days apart, demonstrating the realignment of FOVs after fiber rotation during system reassembly.

(F). Acquisitions of the same FOV and wS1 preparation taken up to 30 days apart, realigned, and compared to track individual neurons across sessions. FOVs are identified based on blood vessels (outlined in red) prior to cells identifications (arrows).

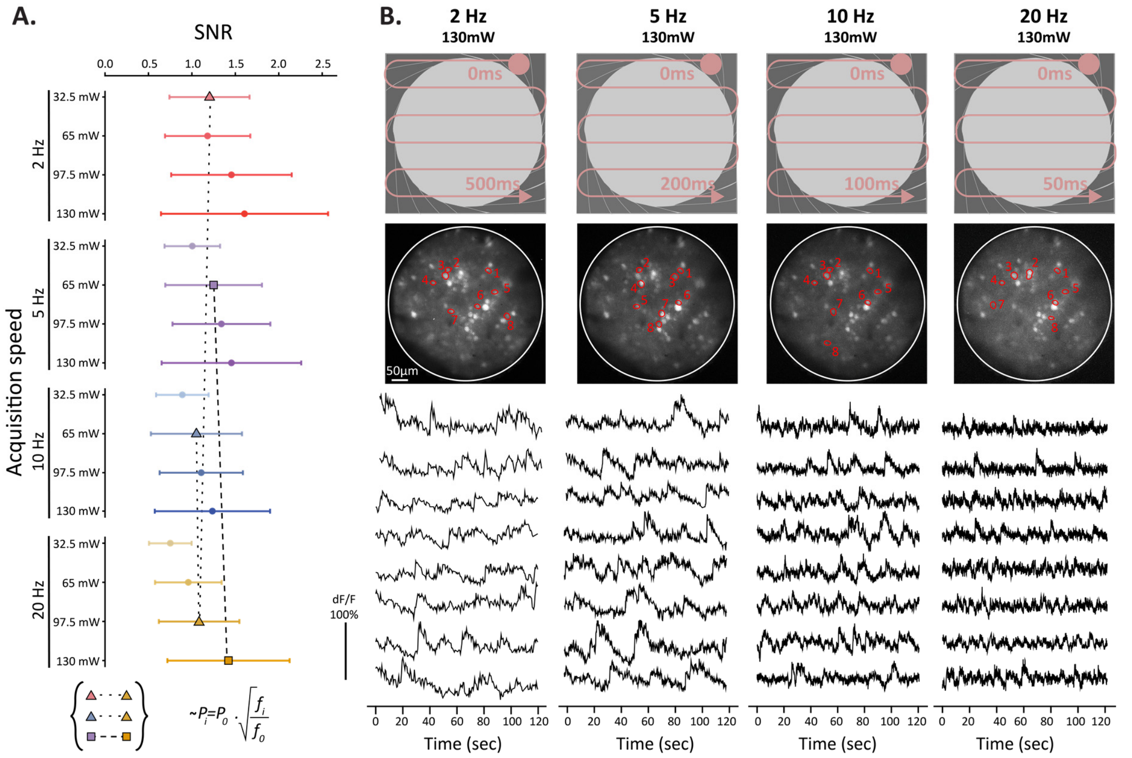

Figure S4: Supplementary data on the maximum achievable imaging speed on a 480µm circular FOV, related to Figure 2.

(A). Imaging acquisitions up to 20Hz with 2P-FENDO-II. Standard deviation of the SNR on a single FOV in wS1 as function of imaging power, 32.5/65/97.5 and 130mW for a scanning spot on the sample plane, and acquisition frame rate for 2Hz (red), 5Hz (purple), 10Hz (blue), and 20Hz (yellow).

(B). Example acquisitions over the same FOV at 2/5/10 and 20Hz for 130mW of laser scanning power. Schemes illustrate the speed of scanning.

Supplemental references

*S*1. Horton, N.G., Wang, K., Kobat, D., Clark, C.G., Wise, F.W., Schaffer, C.B., and Xu, C. (2013). In vivo three-photon microscopy of subcortical structures within an intact mouse brain. Nat Photonics *7*, 205–209. https://doi.org/10.1038/nphoton.2012.336.
