# Supplementary material for: 2P-FENDO-II: A fiber bundle microscope for all-optical, large field-of-view brain studies in freely moving mice
Source: Cell Rep Methods. 2026 Feb 13;6(2):101305. doi: 10.1016/j.crmeth.2026.101305 (PMC12946751; doi:10.1016/j.crmeth.2026.101305)
Supplement: Document S2. Article plus supplemental information [file mmc5.pdf]

Cell Reports
Methods

Article

2P-FENDO-II: A fiber bundle microscope for all-optical, large field-of-view brain studies in freely moving mice

Graphical abstract

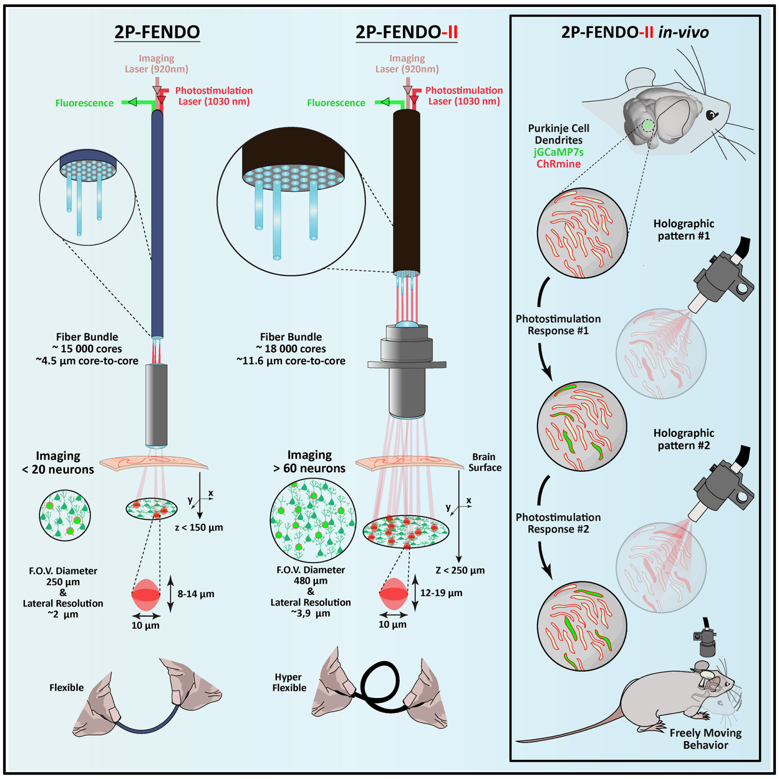

Highlights

- 2P-FENDO-II enables two-photon all-optical study in freely moving mice over a large FOV
- A miniaturized objective reduces optical aberrations
- Leached fibers enhance image homogeneity and flexibility for natural exploration
- We achieved complex shape illumination to target dendrites in freely moving mice

Authors

François G.C. Blot, Dimitri Decombe, Antonio Lorca-Cámara, ..., Benoît C. Forget, Nicolò Accanto, Valentina Emiliani

Correspondence

valentina.emiliani@inserm.fr

In brief

Studying the brain's natural mechanisms requires tools to precisely manipulate widespread neuronal circuits in freely moving animals. Blot et al. present a new microscope that allows the recording and control of neural activity with high precision, down to the level of individual cells, across large brain areas during naturalistic behavior.

Blot et al., 2026, Cell Reports Methods 6, 101305
February 23, 2026 © 2026 The Author(s). Published by Elsevier Inc.
https://doi.org/10.1016/j.crmeth.2026.101305

CellPress

Cell Reports Methods

CellPress
OPEN ACCESS

Article

2P-FENDO-II: A fiber bundle microscope for all-optical, large field-of-view brain studies in freely moving mice

François G.C. Blot,[1,4] Dimitri Decombe,[1,4] Antonio Lorca-Cámara,[1,2] Maya Anquetil,[1] Vincent de Sars,[1] Christophe Tourain,[1] Benoît C. Forget,[1] Nicolò Accanto,[1,3] and Valentina Emiliani[1,5,*]
[1]Institut de la Vision, Sorbonne Université, INSERM, CNRS, 75012 Paris, France
[2]Present address: Kavli Institute for Systems Neuroscience and Centre for Neural Computation, Norwegian University of Science and Technology (NTNU), Trondheim 7491, Norway
[3]Present address: Institute for Bioengineering of Catalonia (IBEC), The Barcelona Institute of Science and Technology, Baldiri Reixac 10-12, 08028 Barcelona, Spain
[4]These authors contributed equally
[5]Lead contact
*Correspondence: valentina.emiliani@inserm.fr
https://doi.org/10.1016/j.crmeth.2026.101305

MOTIVATION Understanding how neuronal activity gives rise to behavior is a central question in neuroscience. Recent advances in miniaturized multiphoton scanning heads have enabled the development of two-photon miniscopes capable of imaging brain activity at cellular resolution during freely moving behavior. While impressive, this technology still lacks the ability to perform all-optical experiments in which neuronal circuits can be simultaneously imaged and manipulated with cellular precision, as is possible under head-fixed conditions. To address this limitation, we previously developed a fiber-bundle-based endoscope, the 2P-FENDO, coupled to GRIN lenses, and demonstrated fast two-photon imaging and optogenetic manipulation in freely moving mice. Here, we present an improved version of the system that achieves a larger and more homogeneous field of view, as well as increased flexibility.

SUMMARY

All-optical strategies enable identification of functional neuronal ensembles with calcium imaging and replay/alter their spatiotemporal activity with optogenetics to decipher their behavioral implications. We previously developed a fiber-coupled microscope enabling two-photon (2P) functional imaging and 2P holographic photostimulation with near-single-cell resolution in freely moving mice: 2P-FENDO. Here, we present a significantly optimized 2P-FENDO-II system that achieves a four-times-larger field of view and a more homogeneous light distribution across the field of view, both for imaging and photostimulation, while achieving better flexibility and thus optimal adaptation to the study of freely moving mice. We demonstrate the performance and versatility of 2P-FENDO-II in experiments targeting the somatosensory cortex, the visual cortex, or the cerebellar cortex, in which we show concomitant calcium imaging with jGCaMP7s and optogenetic control with ChRmine. These enhancements establish 2P-FENDO-II as a groundbreaking tool for all-optical interrogation of neuronal circuits on large volume in naturalistic situations.

INTRODUCTION

Optical-based methods have opened new perspectives in the field of neuroscience to track physiological activity of neurons[1–3] and manipulate[4] arbitrarily chosen ensemble of cells with high spatiotemporal resolution. Imaging techniques have been widely implemented to record with high resolution, at fast frame rate, deep in the brain and on large field of views (FOVs).[5] In parallel, wavefront shaping methods like computer generated holography (CGH)[6] in combination with optogenetics and two-photon microscopy enabled *in vivo* optical control of neuronal activation or inhibition with single-cell and millisecond precision over millimeter-scale volumes. Together, functional imaging and optogenetics have given rise to what is now referred to as all-optical neuronal circuit manipulation, a breakthrough that has driven impressive progress in circuit neuroscience.[4] However, these studies have so far been restricted to head-fixed animals. This limitation has motivated considerable recent efforts to extend all-optical circuit manipulation at cellular resolution to freely moving animals, thereby broadening the study to brain circuits engaged during naturalistic behaviors. The development of miniaturized two-photon miniscopes has enabled fast volumetric

© 2026 The Author(s). Published by Elsevier Inc.
This is an open access article under the CC BY license (http://creativecommons.org/licenses/by/4.0/).

imaging in freely moving rodents at unprecedented spatiotemporal resolution[7–13]; however, these devices remain incompatible with single-cell-resolution-patterned photostimulation. To overcome this limitation, we have very recently developed the first 2P fiberscope capable of simultaneous 2P imaging and holographic 2P photostimulation in freely moving animals, which we named 2P-FENDO.[14] As a multiphoton technique, 2P-FENDO achieves greater depths in the brain and superior axial sectioning for both imaging and photostimulation with respect to previously developed all-optical 1P endoscope.[15,16] 2P-FENDO uses optical fiber bundles composed of many thousands of individual cores to transmit the imaging beam and the holographic patterns for photostimulation to the mouse brain and the emitted fluorescence back to the camera detector. Axially confined transmission of extended excitation patterns was possible, thanks to the inter-core delay dispersion (ICDD), which randomly delays in time the laser pulse transmitted by different cores.[17,18]

Despite its groundbreaking capabilities, the first version of 2P-FENDO had a few limitations. First, the achievable FOV was restricted to a 250-μm-diameter circle, limiting the number of imaged cells. Second, due to laser power, photostimulation was limited to around 10 neurons simultaneously. Third, optical resolution was affected by inhomogeneities in the fiber bundle cores,[19] which led to unwanted image blurring. Lastly, although the type of fiber bundle used was sufficiently flexible to permit freely moving studies, expanding the FOV would require larger stiffer bundles, which would impair the animal's movement.

Here, we address and overcome all these limitations by developing 2P-FENDO-II, allowing high-resolution imaging and precise photostimulation in an FOV of 480 μm, while using highly flexible large-diameter leached fiber bundles, never used before in combination with 2P excitation. Tested over several brain area (V1 cortex, wS1 barrel cortex, cerebellar lobule VI–VII), with neurons expressing jGCaMP7s as indicator and ChRmine as opsin, we show that 2P-FENDO-II is versatile and can address a large range of experimental needs. Being the only system enabling 2P imaging and photostimulation in such large FOV, we believe 2P-FENDO-II will significantly impact behavioral studies in naturalistic settings.

RESULTS

Like our original design,[14] the system is composed of an imaging and a photostimulation path. In the imaging path, a high repetition rate (80 MHz, 130 fs pulse), 920 nm, fiber laser (Alcor, Spark Lasers) is focused to a diffraction limited spot encompassing ~13 different fiber cores and scanned with two perpendicular galvanometric mirrors onto the proximal end of the fiber bundle (simplified scheme in Figure 1A, detailed scheme in Figure S2). The generated fluorescence is collected back through the same fiber bundle and detected by a high sensitivity EM-CCD camera (Andor IXon ULTRA 888). The galvanometric mirrors are configured to scan a squared FOV, which is cropped by an iris to produce a circular scan matching the internal diameter of the fiber (see STAR Methods). Throughout the manuscript, we will refer to the static imaging laser power as the power of a static spot with the galvos fixed to the central position and to the scanning imaging laser power as the average power delivered on the sample while scanning, which, because of the cropping, represents ~65% of the static power.

A low-repetition-rate (1.2 MHz, 420 fs pulse) photostimulation laser at 1,030 nm (NKT) is injected to the fiber in parallel with a dichroic mirror. Single- or multiple-excitation spots are shaped using a liquid crystal spatial light modulator (LC-SLM, Hamamatsu) controlled by a custom-made software WaveFrontV, which also calculates the corresponding holographic phase profiles (see STAR Methods).

The primary goal of 2P-FENDO-II was to enlarge the FOV with respect to what was achieved with 2P-FENDO, so to investigate larger brain areas. To this end, we tested four different combinations of fiber bundles and distal focalizing elements (GRIN lens or mini-objective) as detailed below.

Enlarged FOV and image homogeneity

The achievable FOV (FOV_{max}) depends on the diameter of the fiber (ϕ_{fiber}) and the magnification factor of the optical element on the distal end of the fiber (M), as the fiber diameter is systematically smaller than the focalizing back aperture of the optical element. The original configuration of 2P-FENDO used a fiber bundle (BundleF) made of 15,000 cores (Fujikura, FIGH-15-600N), corresponding to a fiber diameter, ϕ_{fiber}, of 0.550 mm, coupled to a GRIN lens of 2.2x magnification, M_{GRIN}, resulting in a 250 μm FOV_{max} ($FOV_{max} = \frac{\phi fiber}{M} = \frac{0.550}{2.2} = 0.25$, configuration #1; Figures 1B and S1) and a lateral resolution of ~2.0 μm, defined as the inter-core distance (in this case, 4.5 μm) divided by the magnification M_{GRIN}. To increase the achievable FOV_{max}, we screened for commercially available fiber bundles with a ϕ_{fiber} $\geq$ 1 mm. Fujikura offers a range of fiber bundles with larger diameters; however, these are associated with greater stiffness, as indicated by their minimum bending radius (e.g., a bending radius of 30 mm for a fiber with a diameter of 550 μm and 80 mm for a diameter of 1,025 μm), which reduces flexibility and could ultimately constrain the animal's range of motion. To address this, we have turned to a different fiber technology, the so-called leached fiber bundles,[20] in which the outer cladding layer is removed from each core by acid leaching, which renders the bundle extremely flexible (Figure 1B and Table 1).

Here, we have characterized two different leached bundles for imaging/photostimulation experiments, both manufactured by SCHOTT. The first one (SCHOTT IB1010895CTD, named here S-BundleS) has 18,000 cores, a ϕ_{fiber} of 0.9 mm, and an inter-core distance of 8 μm; the second one (SCHOTT IB1651350, named here L-BundleS) has 18,000 cores, a ϕ_{fiber} of 1.45 mm, and an inter-core distance of 11.6 μm. We have coupled the two fibers to a GRIN lens (magnification of 2.2) and a mini-objective (magnification of 3) and compared the achieved FOV and lateral resolution in each case as discussed below and summarized in Table 2.

Coupling the S-BundleS with the same GRIN lens used in our previous publication[14] provided a 410 μm FOV_{max} (configuration #2; Figure S1) and a lateral resolution of ~3.6 μm. However, expanding the FOV also revealed the aberrations of the GRIN lens, notably a significant field curvature effect[21,22] (Figure S1). Specifically, the focal plane difference, Df, between the center and the periphery resulted in 48 μm, as measured using a thin rhodamine layer. We then replaced the GRIN lens by a miniaturized

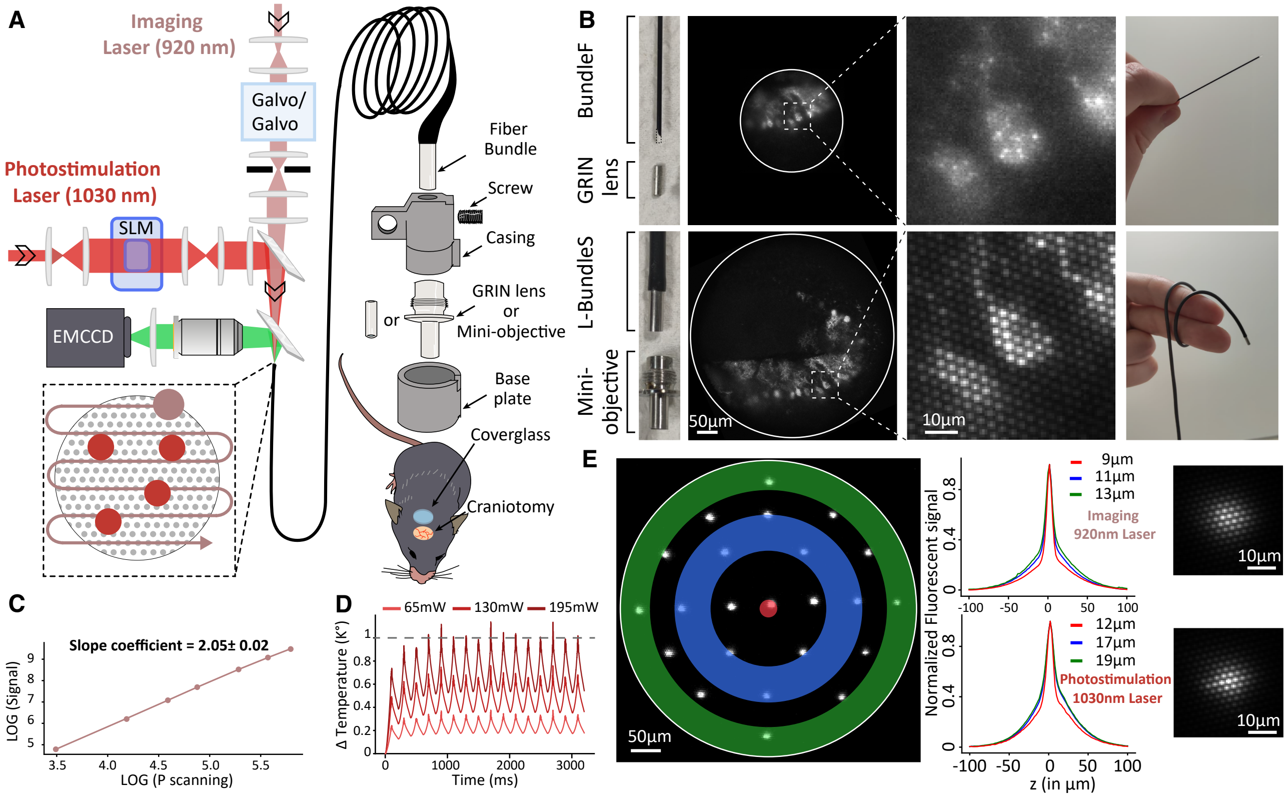

Figure 1. Optical setup and characteristics of the 2P-FENDO-II

(A) Simplified schematic of the optical path for imaging with the 920 nm high repetition laser (130 fs, 80 MHz, 4 W), scanned (pink spot and arrow) with the dual galvanometric mirrors, and photostimulated with the 1,030 nm low repetition laser (420 fs, 1.2 MHz, 54 W), spatially shaped (red spots) by the LC-SLM using computer generated holography (CGH). A zoom on the distal end of the fiber shows the ensemble of elements placed on the animal's head.

(B) Left: image of the Fujikura fiber with the GRIN lens and the SCHOTT fiber with the mini-objective. Middle: image of a 100 μm cerebellar section, with GCaMP6 expressing Purkinje cells with a zoom on one cell showing the resolution obtained with the two configurations #1 and #4. Right: images showing the fiber's flexibility in the two cases.

(C) Log-log plot showing the quadratic dependence of the 2P signal on scanning laser power, measured from illumination of a homogeneous rhodamine layer (powers: 32.5–325 mW). LOG denotes the natural logarithm.

(D) Graph showing the estimated temperature rise over time when scanning with a spot of 10 μm on a circular FOV of 500 μm in diameter at power of 65, 130, and 195 mW (corresponding to a static power of 100, 200, 300 mW) at an acquisition speed of 5 Hz.

(E) Left: fluorescence image of a homogeneous thin rhodamine layer excited by static 1,030 nm spots, with three highlighted distances from the center used to plot the axial resolution for both imaging and photostimulation spot. Center: measured fluorescent axial profiles for spots produced by the imaging and photostimulation lasers in FOV regions corresponding to the color-coded areas in the left image. Right: image of the spots generated by the imaging (top) and photostimulation (bottom) lasers.

objective[23] (3x $M_{objective}$, 3 mm in diameter) corrected for field curvature. The combination S-BundleS + mini-objective (configuration #3) allowed us to observe an almost flat (Df ~4 μm) FOV with diameter of 300 μm and lateral resolution of ~2.6 μm.

Finally, to further increase the FOV_{max}, we replaced the S-BundleS with the larger L-BundleS. This increased the FOV_{max} to 480 μm (configuration #4; Figures 1B and S1), with minimal field curvature (Df ~10 μm) and lateral resolution of ~3.9 μm. Despite the lower resolution achieved with the S- and L-BundleS (configurations #2/3/4), the ability to distinguish individual cell soma or even some proximal dendrites was not compromised (Figure 1B).

Along with an increase in the FOV, we noticed a difference in image homogeneity for both S- and L-BundleS compared to BundleF (Figures 1B and S1).

The FIGH series from Fujikura are manufactured with stochastic inhomogeneity in size and shape of the cores in order to reduce core-to-core cross talk.[19,24] This produces a certain core-to-core excitation inhomogeneity, both in the transmitted intensity and in the wavelength dispersion, which is especially critical for 2P imaging and can reduce overall the quality of images. This effect is less pronounced in the SCHOTT bundles, resulting in sharper images. On the cerebellar section, this improvement allowed, for example, more reliable discrimination of the dark nuclei within the fluorescent somata (see Figures 1B and S1).

Given the expanded FOV and the higher excitation homogeneity, we decided to focus the manuscript on this final configuration for 2P-FENDO-II: L-BundleS combined with the miniaturized objective. Compared to the original 2P-FENDO,

Table 1. Summary of characteristics of three fiber bundles used in the different configuration of the system

Manufacturer reference	Fujikura FIGH-15-600N	SCHOTT IB1010895CTD	SCHOTT IB1651350
Paper designation	BundleF	S-BundleS	L-BundleS
Length (m)	2(used in 2P-FENDO but adaptable)	0.895	1.35
Bending radius (mm)	30	∼5	∼5
Quality area (μm)	550	900	1450
Number of cores	15,000	18,000	18,000
Inter-core distance (μm)	4.5	8.0	11.6
Inter-core homogeneity	variable	constant	constant

2P-FENDO-II in this configuration doubles the FOV diameter from 250 to 480 μm, thereby reaching a more than 4-fold increase in both accessible FOV area and number of detectable cells.

Two-photon efficiency and resolution of 2P-FENDO-II

The 2P illumination efficiency through optical fibers can be affected by non-linear effects, such as the self-phase modulation (SPM),[25] which induces a significant pulse broadening when the power per core exceeds a defined power threshold corresponding to a pulse broadening, which was experimentally found to be ∼10 mW/core for 920 nm, ∼100 fs, and 80 MHz.[25] The two-photon fluorescence signal (*Fl*) depends on the peak power of the laser (P), the repetition rate (f), and the pulse width (τ), as described by the relationship $Fl \propto \frac{P^2}{f\tau}$. To investigate potential SPM in the L-Bundle at 920 nm, we measured the 2P fluorescence (*Fl*) induced on a rhodamine layer while scanning a spot of ∼10 mm in diameter (corresponding to ∼13 cores; see STAR Methods). Since Fl depends on peak power (P), repetition rate (f), and pulse width (τ) according to $\propto \frac{P^2}{f\tau}$, we tested the signal power dependence for a range of scanning input powers (32.5–325 mW). Our experimental results revealed a quadratic relationship between *Fl* and the input laser power, indicating that τ remains constant within the tested power range. This confirms that we always operated below the threshold for significant SPM (Figure 1C).

High-energy two-photon laser scanning can induce significant temperature increase of the tissue,[26,27] which, if exceeding a few degrees, can alter physiological activity[28–30] and even lead to cell death.[31] This effect may be further exacerbated in our configuration, where a larger scanning spot is used compared to conventional two-photon microscopy, thereby reducing the efficiency of local heat dissipation. To estimate the temperature rise induced under our imaging conditions, we extended a thermal model previously developed to evaluate temperature changes under optogenetic stimulation.[26] Specifically, we simulated the temperature evolution at a single central spot in the FOV (see STAR Methods) during scanning of a 500-μm-diameter area at increasing laser power and an acquisition rate of 5 Hz (Figure 1D). In agreement with previous findings,[27] the model predicts temperature peaks exceeding 1°C when scanning at 195 mW, whereas no such increase is observed at 65 or 130 mW. Based on these results, we limited the scanning power to ≤130 mW (corresponding to ≤5 mW/core; see STAR Methods) in all subsequent experiments to remain below the 1°C safety threshold.

For both imaging and photostimulation, we used 10-μm (full width half maximum [FWHM]; corresponding to ∼13 illuminated cores; see STAR Methods) diameter spots. The optical axial resolution was 9 μm at the center of the FOV, increasing to 13 μm at the edges, for the imaging laser, and 12 μm at the center, increasing to 19 μm at the edges, for the photostimulation laser (Figure 1E). The difference in axial resolution between the two lasers is likely to be attributable to the longer pulse duration of the photostimulation laser (420 fs) compared to the imaging laser (130 fs), which partially counteracts the effect of the ICDD.

As previously observed,[32,33] these values remained well preserved at the working depths used in the *in vivo* experiments (see Figure S2; STAR Methods).

In vivo two-photon imaging in freely moving animals with 2P-FENDO-II

To test the imaging quality of the 2P-FENDO-II, we performed experiments on several brain regions in freely moving animals: the visual cortex (V1, 2 mice), the barrel cortex (wS1, 3 mice), and the cerebellar cortex in the vermis of lobule VI–VII (CbC, three mice) (Figure 2). The long working distance of the mini-objective (1 mm) allows imaging of very deep layers. However, scattering, combined with camera detection, limited the maximum imaging depth to approximately 250 μm when visualizing blood vessels following periorbital injection of fluorescein (Figure S3A) and to 160 μm when recording calcium signals in the cortex and cerebellum in animals injected with jGCaMP7s (Figure S3B).

The fiber and the mini-objective were joined, aligned, and protected by a custom-made casing adapted from the design of Zong et al.,[23] 3D printed in resin, where the mini-objective was clipped and the fiber maintained in position with a lateral screw in contact with the metal coating (Figure 1A). Once the base plate was cemented onto the animal's head, the position of the mini-objective relative to the cover glass could not be easily adjusted. However, manual adjustment of the fiber relative to the mini-objective allowed repositioning of the imaging focal plane. As shown in Figure S3C and Video S3, this approach enabled imaging of either dendrites (at 50–90 μm depth) or somata (at ∼160 μm depth) of Purkinje cells in the same animal. This adjustment required head-fixing the animal and could not be performed accurately during free behavior. Additionally, manipulating the fiber within its casing could cause it to rotate, leading to a corresponding rotation of the FOV on the camera (Figure S3). However, once the lateral screw was re-secured, stabilizing the fiber's distal end, no further FOV rotation or focal drift was observed during the same day of acquisition. To assess whether the same cells could be reliably tracked across extended periods (days to weeks), we manually realigned images post hoc, to correct for FOV rotation. Using this method,

Table 2. 2P-FENDO configuration characteristics

Configuration	Fiber bundle	Focal element	Lateral resolution	FOV_{max}	Field Curvature (focal difference at max FOV)
#1 (2P-FENDO)	BundleF	GRIN	2.0	250 μm	21 μm
#2	S-BundleS	GRIN	3.6	410 μm	48 μm
#3	S-BundleS	MiniObj.	2.6	330 μm	4 μm
#4 (2P-FENDO-II)	L-BundleS	MiniObj.	3.9	480 μm	10 μm

Theoretical values of the *Lateral resolution* $= \frac{inter\ -\ core\ distance}{M}$; $FOV_{max} = \frac{\phi fiber}{M}$ for the different configuration of the system and experimentally measured value for the field curvature, for four different configurations defined by the first two columns. Configuration #4 highlighted in gray is the one adopted in the manuscript.

we successfully aligned FOVs acquired weeks apart in the same animal (Figures S3E and S3F). Blood vessels served as stable anatomical landmark, allowing accurate alignment and long-term tracking of individual cells.

The casing (0.43 g) + mini-objective (0.6 g) + base plate (0.29 g) weighted together 1.32g. The L-BundleS itself weighted 9.2 g, and this weight was supported by two helium balloons, each providing an estimated buoyant lift of up to 12 g each.[34]

We tested whether the novel fiber and the total weight of the system affect the animal's range of movement using video recordings in the animal's home cage, both with and without the 2P-FENDO-II. Animals were recorded under red light, which was completely filtered out on the collection path before reaching the EM-CCD camera (Figure 2A; Video S1). DeepLabCut analysis revealed same exploration rate (*p= 0.1*) and probability distribution of speed when tested with and without the 2P-FENDO-II (Figure 2A). These results demonstrated that neither the weight of the holder nor the fiber affects the animal's exploration behavior.

We tested the dependency of the signal on the imaging laser power (P_i = 200, 150, 100, and 50 mW, measured for a static spot at the exit of the mini-objective, corresponding to a scanning power of 130, 97.5, 65, and 32.5 mW) and acquisition frame rate (f_i = 2, 5, and 10 Hz), maintaining the same excitation spot size (10 μm FWHM; Figure 2C) and a power/core below the threshold for SPM-induced pulse broadening (Figure 1C), imaging wS1. Using CaImAn analysis,[35] the recorded images were processed for motion correction, cell segmentation, SNR calculation, and traces extraction. We set a threshold of SNR>2, to extract clearly responding cells over all the detected components. As expected, optimizing the SNR requires finding a compromise among excitation power and acquisition frame rate (Figure 2C). Specifically, to maintain a comparable SNR, at constant spot size when changing the acquisition frame rate from f_0 to f_i, the imaging power must be increased to $\sim P_i = P_0 \cdot \sqrt{\frac{f_i}{f_0}}$.

In all regions, we demonstrated calcium transients at 2 Hz and 10 Hz imaging frame rates on the enlarged FOV of the 2P-FENDO-II using an illumination scanning power of 130 mW (Figure 2D; Video S1). In both V1 and wS1, somata from layer 2/3 neurons were recorded at a depth varying from 90 to 120 μm from the brain surface. In the cerebellum, Purkinje cell (PC) dendritic arborizations were imaged in the molecular layer at a depth of 50 to 70 μm. The fast temporal dynamic (50–120 Hz) of simple spike PCs cannot properly be revealed with calcium imaging at the soma[36]; meanwhile, their complex spike activity (1–2 Hz) could be acquired at the dendrite level with calcium indicators.[37]

Exemplary data on three different animals confirmed our capability of recording calcium transients from several tens of neurons with high SNR. Precisely, at an acquisition rate of 2 Hz on the example FOVs, we measured 48 components with SNR ≥2 over 106 total components for V1, 62/94 for wS1, and 42/61 for CbC (Figure 2D). At an acquisition rate of 10 Hz using the same imaging power, we could record 79 components with SNR ≥2 over 105 total components for V1, 61/117 for wS1, and 28/39 for CbC (Video S1).

To further test the sampling rate, we performed acquisitions at 20 Hz in the cortical area wS1 (Figure S4). We showed that at all power tested, the SNR remains comparable to the one achievable at a slower rate, f, when powers were increased proportionally to the expected scaling factor: $\sqrt{\frac{f_i}{f_0}}$.

In vivo two-photon imaging and holographic photostimulation in freely moving mice with 2P-FENDO-II

We tested the patterned photostimulation with the 2P-FENDO-II on both cortex (wS1, 4 mice) and cerebellum (CbC, 3 mice). Viral injections of AAV1-hSyn-ChRmine-mScarlet-Kv2.1-WPRE together with AAV9-*syn*-jGCaMP7s-WPRE (ratio 1:2) performed at the time of the cranial window surgery were done on either one of these two regions one month prior to the acquisitions. In both wS1 and CbC preparations, we observed co-expressing cells together with an imperfect soma targeting of the ChRmine, which, as previously observed, leads to opsin expression in proximal dendrites, as confirmed by postmortem histological analysis (Figure 3A). In some cells, this also extended to distal dendritic segments.

We tested three scanning imaging laser powers (65, 97.5, and 130 mW) and observed significant crosstalk due to opsin activation by the 920 nm laser when the power exceeded 97.5 mW (Figure 3B). The initial increase in activation at the onset of acquisition was not observed when jGCaMP7s was expressed alone, even at the highest laser power tested (130 mW). This indicates that, consistent with our modeled temperature changes (Figure 1D), the effect did not result from thermal damage induced by the imaging laser, or by non-linear damage, but rather reflects clear opsin cross-activation by the imaging laser.

To minimize this crosstalk, we set a maximum scanning imaging laser output of 65 mW for subsequent experiments in which ChRmine and jGCaMP7s were co-expressed.

On each experimental day, an image was acquired at the beginning of the session to define the reference position of the cells in the FOV. Loaded onto WaveFrontV,[38] this allowed to position the photostimulation spots and the calculation and

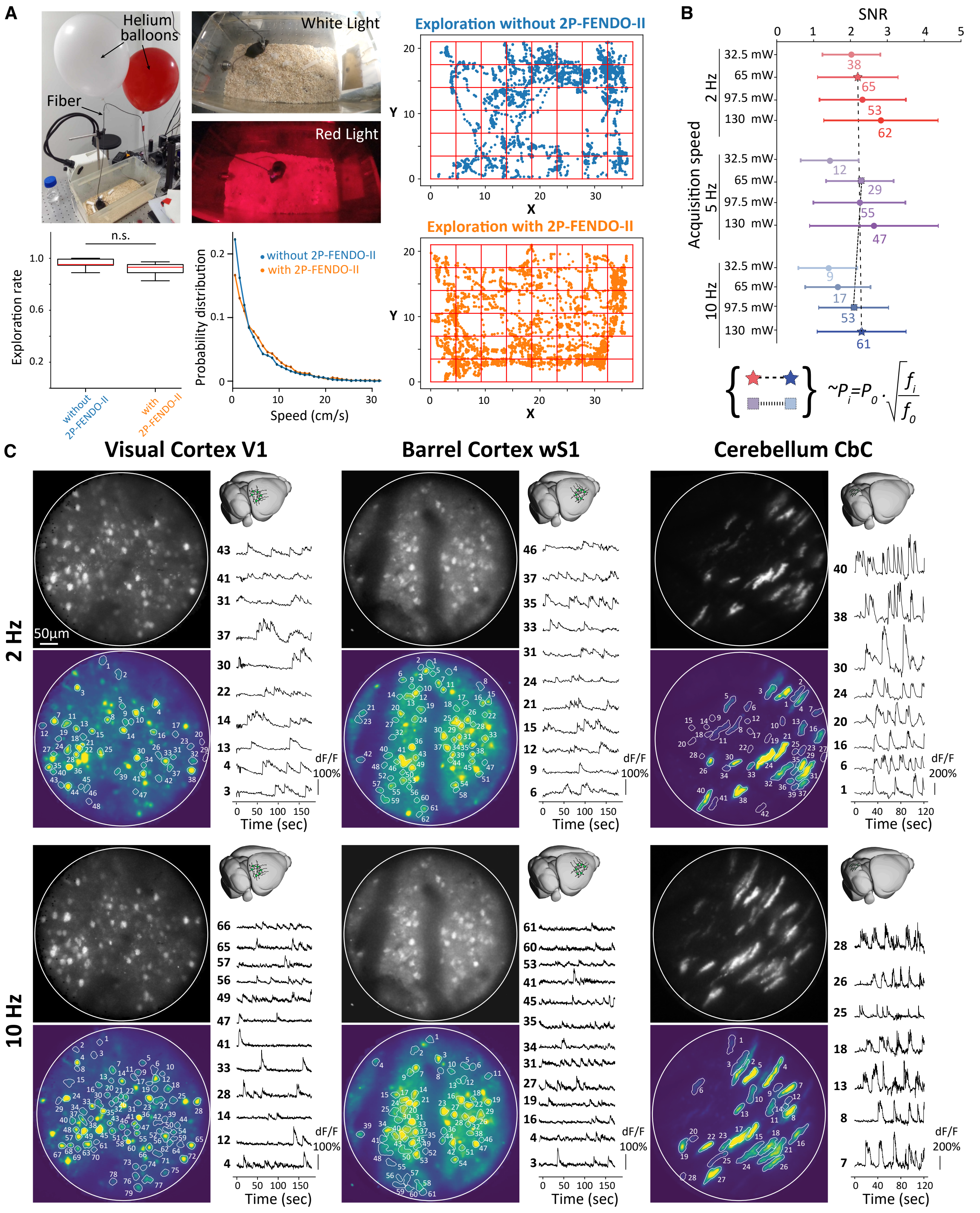

(legend on next page)

generation of the phase profile, while applying the correction for the position-dependent diffraction efficiency of the LC-SLM and the optical aberrations and geometric distortions of the system. Compared to the original 2P-FENDO, generated holographic spots to target cell's somata are replaced by Gaussian spots multiplexed by the LC-SLM. The strategy prevents speckles, intrinsic to holographic spots, which could be particularly problematic when using the large inter-core distance of S-Bundle. We demonstrate photostimulation of ensembles of 10 (patterns #1 and #2, Figure 3C; Video S2) to 24 neurons (pattern #3) using 10 μm FWHM multiplexed Gaussian spots with an average power of 30 mW (~1.15 mW per core; see STAR Methods) per spot. Traces of 28 cells in the FOV were extracted to illustrate the response of targeted and responding cells (T/R, *red*), targeted and non-responding cells (T/nR, *black with red asterisk*), all non-targeted but responding cells (nT/R, *gray*), and few examples of non-targeted and non-responding cells (nT/nR, *black*). Patterns #1 and #2 were acquired on the same experimental session, while pattern #3 was acquired a week later on a similar FOV. Therefore, the 28 cells targeted with pattern #1/2 were not exactly the same 28 ones targeted with pattern #3. The photostimulation protocol consisted of a train of 10 pulses, each of 20 ms duration, delivered at a frequency of 4 Hz, as defined by the camera acquisition settings. Specifically, the camera was kept ON for 200 ms intercalated with 50 ms of camera OFF between each frame. During these OFF periods, the photostimulation laser was sent to the sample. This avoided the collection of artifactual signals generated by direct fluorescence and fiber autofluorescence excitation from the 1,030 nm laser (Figure 3C, bottom inset). During each acquisition, the photostimulation paradigm was repeated four times (Figure 3C, vertical red lines), with intervals of tens of seconds, to better distinguish the photostimulation response from spontaneous activity events.

Identification of photostimulated cells was performed using CaImAn,[35] with a custom-made cell-sorting method. The responding target cells were defined as those having an average response in the 2 s following the photostimulation at least three times larger than the standard deviation of dF/F in a time window of 2 s before the onset of the photostimulations. In pattern #1, we found 10/10 T/R cells, 4 nT/R cells, and 14 nT/nR cells. In pattern #2, we found 10/10 T/R cells, 5 nT/R cells, and 13 plotted nT/nR. In pattern #3, we found 22/24 T/R cells, 2/4 T/nR cells, 3 nT/R cells, and 1 nT/nR cell. Nontargeted but responding cells were always found in proximity of a targeted cell. This spurious activation can be due to a non-perfect selectivity of our photostimulation laser or imperfect soma targeting of the opsin, as it is apparent in Figure 3A. As we see dendritic expression of the opsin until few tens of μm from the soma, it is possible that, while targeting the soma of one cell, we also excite dendrites of a close-by cell.

To statistically quantify off-target photostimulation probability, we plotted for all the acquisitions (separated in three mice) the percentage response of dF/F of detected cells in the FOVs and the overall photostimulation probability (averaged on all acquisitions and mice) as a function of their distance from the closest photostimulation spot (Figure 3D). A fit of the photostimulation probability with an exponential decay function (see STAR Methods) gave a decay constant of 18.4 ± 0.3 μm, in agreement with our previous publication.[14]

In the cerebellar preparation, we took advantage of the dendritic localization of the opsin and the high excitability of ChRmine. We set the imaging plane onto PC dendrites in the molecular layer, more suitable to follow calcium transients, while precisely photostimulating the same plane. We leveraged the capability of CGH to create excitation patterns of arbitrary shapes,[38,39] enabling precise targeting of dendritic arborizations in CbCs with varying lengths and shapes (Figure 3E; Video S2). Using WaveFrontV, we generated these patterns based on fluorescence images and automatically adjusted the laser power for each shape. This compensation ensured uniform excitation density (or power per unit area) despite variations in the excitation area. With the 2P-FENDO-II, we demonstrated the ability to independently target neighboring dendritic structures and reproduce calcium spikes that closely mimic the physiological complex spikes observed in cerebellar Purkinje cells (Figure 3E; Video S2).

In the original 2P-FENDO system, a 5 W Goji laser (1,040 nm central wavelength, 150 fs pulse duration, 10 MHz repetition rate) delivered up to ~300 mW to the sample, after accounting for optical and fiber losses. Since the power was evenly split across stimulation spots, when using 30 mW per spot, the set up could target approximately 10 cells simultaneously. In contrast, the upgraded 2P-FENDO-II system uses a 54 W NKT laser, which removes previous limitations in power delivery on the achievable number of targets. Under these conditions, thermal heating[26] is the only limitation on the number of targets that can be stimulated simultaneously.

DISCUSSION

We have demonstrated a holographic endoscope designed for large FOV 2P-imaging and 2P-photostimulation at cellular

Figure 2. Two-photon calcium imaging on large, 480-μm FOVs in the visual (V1), barrel (wS1), or cerebellar (CbC) cortex in freely moving animals with 2P-FENDO-II

(A) Photos of the animal in the cage and DeepLabCut tracking of animal movements in the home cage with or without the 2P-FENDO-II. Graph: exploration rate (left) and probability distribution of mouse speed (right) using DeepLabCut tracking for two animals in their home cage, with (orange) and without (blue) the 2P-FENDO-II.

(B) Evolution of the SNR on a single FOV in wS1 as function of scanning imaging power, 32.5/65/97.5 and 130 mW, and acquisition frame rate of 2 Hz (red), 5 Hz (purple), and 10 Hz (blue). Numbers indicate the number of components with SNR>2.

(C). Images of z-projection of non-processed data (black & white, see Video S1) and projection of processed images for motion correction and smoothing (yellow & blue) from CaImAn analysis, with detected components with SNR >2 with the schematic representation of the injected location of AAV9-Syn-jGCaMP7s-WPRE and few examples of calcium transients' detected at 2 Hz (top) or 10 Hz (bottom) imaging frame rate and scanning imaging power around 130mW. Acquisition in V1 and wS1 are done in layer II/III at 90 to 120 μm at intervals of 4 and 30 min respectively and in the molecular layer of CbC at 70 μm deep at 2 days interval.

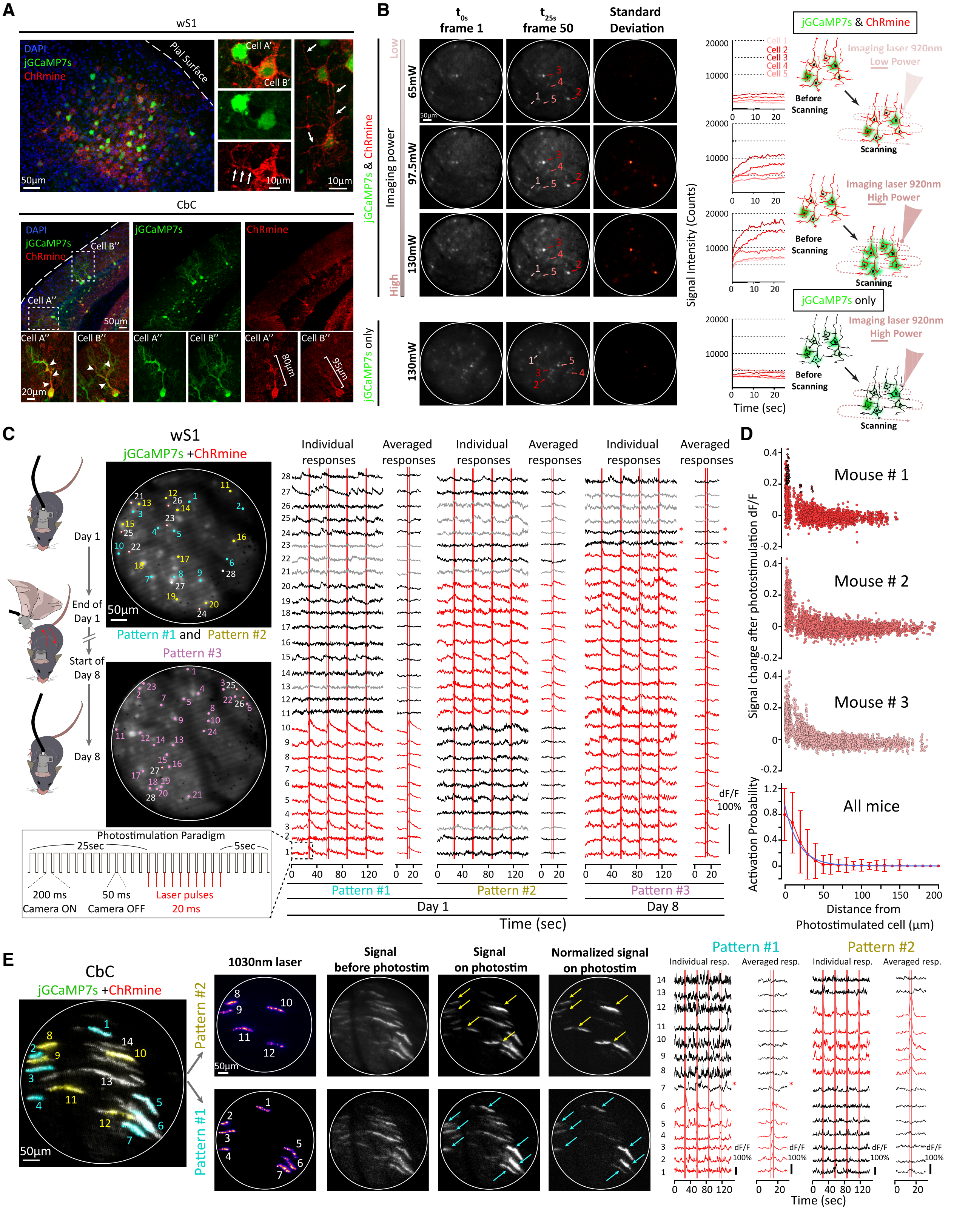

(legend on next page)

CellPress
OPEN ACCESS

resolution, the 2P-FENDO-II. We tested several configurations of the system using leached SCHOTT fibers of two different diameters (S-BundleS and L-BundleS), paired with either a 1 mm diameter GRIN lens or a 3 mm diameter mini-objective to compare with our previous design of the 2P-FENDO.[14] While leached fiber bundles have been previously studied and characterized,[40,41] to the best of our knowledge, this work represents the first successful demonstration of two-photon excitation using leached fiber bundles. The larger inter-core distance of the tested SCHOTT fibers led to a correspondingly lower lateral resolution compared to that achievable with Fujikura fibers. Nevertheless, the lower nominal resolution of the SCHOTT did not compromise the ability to differentiate cell bodies and observe some dendritic processes. Moreover, the higher core homogeneity of SCHOTT fibers, compared to the previously used Fujikura fibers, resulted in improved imaging quality due to more uniform core-to-core excitation (configurations #2/3/4). Meanwhile, the axial confinement was successfully maintained at 9–13 μm with the new fibers (tested for Configuration #4). The leaching process employed for SCHOTT fiber bundles imparts remarkable flexibility, allowing for a wide range of motion for the animal. This flexibility positions these fibers as a promising solution for the next-generation, holographic endoscopes.

We demonstrated that configuration #4, which utilizes an L-Bundle paired with the 3 mm mini-objective, achieves the largest FOV with minimal curvature aberrations. This configuration, named 2P-FENDO-II, is therefore the optimal choice for investigating large neuronal networks in superficial cortical regions. Meanwhile, configurations employing the 1.3-mm-diameter GRIN lens (configurations #1 and #2) are better suited for deep brain imaging. Configurations #3 and #4 could also be considered for deep brain structure imaging through the coupling of the mini-objective with a GRIN lens as previously done by Zong et al.[23] SOTA for multiphoton miniscope imaging system, the MINI2P achieves an FOV_{max} of 550 μm with a lateral resolution of 1.15 μm for a z resolution of 18 μm and demonstrated imaging speed of 15 Hz. The 2P-FENDO-II achieves an FOV_{max} of 480 μm with a lateral resolution of 3.9 μm for a z resolution of 9–13 μm and demonstrated imaging speed of 20 Hz and enables simultaneous optogenetic stimulation. We have validated the capability of 2P-FENDO-II for 2P imaging in freely moving animals in three different brain regions including the visual cortex, the barrel cortex, and the cerebellar cortex at depths ranging from 50 to 160 μm. The configuration enabled us to perform calcium imaging at up to 20 Hz acquisition rates while maintaining a high-quality SNR. We can vary the acquisition frame rate while maintaining a constant SNR by keeping a spot size of 10 μm FWHM and proportionally increasing the imaging power, ensuring that the power per core remained below the threshold for SPM-induced pulse broadening.[25,42]

Compared to the MINI2P, which represents the SOTA for 2P imaging in freely moving animals, the 2P-FENDO-II system exhibits lower lateral resolution and acquisition speed and, in its present configuration, does not include a tunable lens for multiplane imaging (see Table 3). However, it features a more compact design by eliminating the need for MEMS-based scanning and associated wiring at the animal's head. The key innovation of this new system lies in its capability for holographic photostimulation, a functionality not available in the MINI2P (but see Futia et al.[43] for a related approach), which can now be implemented over a significantly larger FOV compared to the previous generation of 2P-FENDO.

We demonstrate that the 2P-FENDO-II system outperforms our original 2P-FENDO in several key aspects, including image homogeneity, fiber flexibility, imaging depth, FOV size, field curvature, and the number of accessible cells, all while maintaining the frame rate, resolution standards, and overall system weight.

Figure 3. Two-photon calcium imaging and holographic photostimulation with 2P-FENDO-II in freely moving animals injected with AAV9-Syn-jGCaMP7s-WPRE and AAV1-hSyn-ChRmine-mScarlet-Kv2.1-WPRE

(A) Confocal microscopy images of sagittal sections from injected mice in wS1 or CbC. Co-expression is observed in cortical wS1 cells with ChRmine-mScarlet protein observed in somata and dendritic arborizations. Cells A′ and B′ illustrate entanglement of a ChRmine-positive proximal dendrite around a neighboring soma. In the Cbc, the expression of ChRmine-mScarlet in PCs extends from the somata to distal segments of the primary dendrites as pointed for cells A'' and B'' (white arrow heads).

(B) Scheme of the crosstalk effect of activation by the imaging laser. Image of the first frame (left column) and the fiftieth frame (middle column) for different scanning power, 65/97.5 and 130 mW from the 920 nm imaging laser. These parameters were tested on brains expressing either jGCaMP7s + ChRmine or jGCaMP7s alone. Standard deviation of the two frames (right column) illustrating the relative change of signal during the first 25 s of acquisition. Traces of signal counts for five selected cells are plotted to illustrate crosstalk activation.

(C) Example FOVs over the wS1 cortex (~110 μm deep) taken on two different days at scanning imaging power of 65 mW. The photostimulation and camera frame paradigm is illustrated in the rectangle. The paradigm is repeated four times during a single acquisition (vertical lines). For each FOV, calcium transients (dF/F) of 28 cells are plotted among which either 10 or 24 are targeted for photostimulation with three different patterns: pattern #1 targets cells #1–10 on the FOV of day 1 (upper image), pattern #2 targets cells #11–20 on the FOV of day 1 (upper image), and pattern #3 targets cells #1–24 on the FOV of day 8 (lower image), with 30 mW of average power per cell for each pattern. Colored dots point targeted cells, gray dots circled in red point for all non-targeted cells presenting responses to the photostimulation, and whole gray dotes point example cells that are non-targeted and present no responses. Individual responses and averaged responses in the time window of the photostimulations are shown in red for targeted and responding cells (T/R), in black for selected examples of non-targeted and non-responding cells (nT/nR), in gray for all non-targeted but responding cells (nT/R) detected in the FOV, and in black with a red asterisk for targeted but non-responding cells (T/nR).

(D) Plot of the change of response of individual cells, in three mice, within the photostimulation window in function of the distance from the closest photostimulation spot. Activation probability of neurons as a function of the distance from the photostimulated cells with an exponential decay (blue curve).

(E) Example FOV in CbC (~90 μm deep) vermal lobule VI. Dendrite-shaped holograms are generated to target cells of either pattern #1 (cyan, 7 dendrites) or pattern #2 (yellow, 5 dendrites). Acquisition with simultaneous camera ON and photostimulation laser ON was done to image the holograms ("1,030 nm laser") prior to the photostimulation experiments. For each acquisition, a maximum projection of all images acquired around the photostimulation window ("Signal before photostimulation") and of four images (for the four repetitions of the paradigm in an acquisition) at the peak of photo-activation ("Signal on photostim") were subtracted from one to another to obtain the "Normalized signal on photostim" and show the cells specifically responding to all the photostimulations.

Table 3. Two-photon miniature microscope characteristics

	MINI2P	M-MINI2P	2P-FENDO	2P-FENDO-II
Weight	2.4 g	<3 g	0.7 g	1.32 g
Remote focusing	yes (axial scan of 240 μm; weight 60 mg)	yes (axial scan of 230 μm)	none	none
Max FOV	500 μm, square (MINI2P-L, single FOV) 420 μm, square (MINI2P-F, single FOV)	500 μm, square	250 μm, circular	480 μm, circular
Scanning	MEMS	MEMS	Galvo – resonant	Galvo – Galvo
Speed	15 Hz (MINI2P-L, 256 line) 40 Hz (MINI2P-F, 256 line)	81.5 Hz (256 × 256 pixels) stitching 2 channels	50 Hz	20 Hz
Detection	benchtop PMT,flexible fiber	benchtop PMT, flexible fiber	benchtop camera, fiber bundle	benchtop camera, fiber bundle
Optical imaging resolution	lateral 1.2 μm axial 13 to 18 μm	on-axis/off-axis lateral 1.37/1.34 μm axial 23/24 μm	lateral 2.0 μm axial 7 to 13 μm	lateral 3.9 μm axial 9 to 13 μm
Demonstrated imaging depth GCamp signal	not specified, cortical layer 2/3	230 μm	150 μm	160 μm
Holographic single-cell photostimulation	none	none	yes	yes
Demonstrated stimulated cell	0	0	10	21

Comparison between 2P-FENDO-II, 2P-FENDO, Mini2P and M-Mini2P (Zhang et al.[13]) in imaging and photostimulation capabilities. Adapted from Klioutchnikov and Kerr.[44]

A major limitation of the original 2P-FENDO system was the short working distance (200 μm) of its GRIN lens, which restricted imaging depth to about 150 μm below the cover glass. In contrast, 2P-FENDO-II integrates a miniaturized objective with a 1 mm working distance, allowing imaging at depths now primarily limited by tissue scattering and camera detection (typically ~160 μm below the cover glass for Ca imaging and up to ~250 μm for blood vessels imaging). Furthermore, the enlarged FOV yields a substantial gain in the number of detectable cells. Under identical imaging conditions (2 Hz, 135 mW) in the same brain region using the same indicator, the original 2P-FENDO system detected ~15 cells with SNR ≥2, whereas 2P-FENDO-II detected ~62 cells, demonstrating a 4-fold increase in the achievable number of targets. This represents a critical advancement for the interrogation of neural networks. While previous holographic optogenetic studies have shown that selective activation of small neuronal ensembles (<30 cells) can drive behavior,[45–47] these findings also underscore that network output depends on the functional motifs rather than the absolute number of neurons. The identification of such behaviorally relevant ensembles is inherently constrained by the imaging surface area, as functional motifs frequently extend over hundreds of microns to millimeter scales. By doubling our accessible FOV (from 250 to 480 μm), 2P-FENDO-II now enables enlarging the field for a systematic exploration of these spatially distributed networks in freely moving animals.

We demonstrated the capability of the 2P-FENDO-II to selectively photostimulate neurons at near-single-cell resolution using either simple spots or complex shapes of illumination. All-optical experiments were conducted using mice co-expressing ChRmine and jGCaMP7s. In these experiments, the imaging power was kept below 65mW to minimize artifactual crosstalk activation of the opsin by the imaging laser. Higher imaging power—and consequently improved image quality—could be achieved by minimizing imaging cross-talk. This can be achieved by using faster opsins,[48] more red-shifted opsins,[49] blue-shifted calcium indicators with 800 nm imaging lasers,[50] and/or by further increasing the imaging speed.[48]

Hybrid solutions that combine two-photon (2P) miniscopes for imaging with holographic fiber bundle excitation[51,52] could offer a balanced approach, optimizing imaging lateral resolution and collection efficiency while preserving the capability for patterned stimulation. Already in development,[43] such strategies may introduce additional constrains such as increase in volume and weight. In contrast, the lightweight design of 2P-FENDO-II, at just 1.32 g, offers a significant advantage for studying naturalistic behavior.

It is worth noting that all 2P-FENDO configurations, including the one demonstrated in this work, can be readily integrated with any commercial two-photon imaging or holography system by replacing the conventional high-NA objective with a low-numerical-aperture one, which couples the scanning or holographic beams into the fiber bundle entrance. By adjusting the distance between the imaging or holographic focal plane and the fiber bundle entrance, the size and position of the imaging or photostimulation spot can be tuned, thereby optimizing imaging speed or photostimulation efficiency. The adapters described in

the STAR Methods provides accurate alignment and stable mechanical coupling of the fiber bundle to the GRIN lens or mini-objective.

While we have learned to differentiate brain activity among sleep, anesthetized, and awake states, several studies now highlight the need to differentiate awake restrained and awake freely moving as distinct brain states.[53] Circuit recruitment and cellular computation are significantly influenced by head and/or body restrictions, which can lead to artifactual processing. Recent studies have demonstrated drastic changes in cerebellar activity[54] and change in neurons recruitment in the hippocampus[55,56] when animals navigate in three-dimensional environments. Although portable imaging tools have seen extensive development, systems for selective neuronal activation in freely moving rodents are emerging and are predicted to offer unlimited perspectives for the field.

Limitations of the study

We demonstrated 2P calcium imaging at up to 20 Hz over a ~500 mm FOV. Further increasing the acquisition rate is challenging with the current system, as the galvanometric mirrors approach their speed limit and the EMCCD camera experiences thermal limitations at higher frame rates for such a large imaging area. These limitations could be addressed by replacing the galvo-galvo configuration with a galvo-resonant system, to increase scanning speed and therefore sampling rate and by replacing the EMCCD with a faster CMOS camera. Increasing or multiplexing the excitation spot also represents a viable solution to further increase the acquisition speed.

We demonstrated that stable attachment of the system to the mouse's head, combined with the brain's limited intracranial motion, allows reliable tracking of the same cells over several hours. Over longer intervals, while lateral brain displacement (x and y axes) was minimal, reliably recovering the exact same FOV remained challenging due to two main factors: (1) potential rotation of the FOV caused by distal fiber tip rotation during handling and (2) difficulty in reproducing the original focal plane. FOV rotation can be detected and corrected post-hoc, while focal plane adjustments are achieved by manually shifting the fiber bundle relative to the mini-objective, enabling reliable recovery of the same plane. Future integration of micro-tunable lenses at the fiber's distal end could further improve z-plane positioning precision.

For longitudinal recordings spanning months, the most limiting factor was the fading of GCaMP transients from AAV-mediated expression, typically 2–3 months post-infection. This limitation could be addressed by using transgenic mouse lines.[57,58]

The performance of fiber-bundle-based endoscopes, here demonstrated in combination with commercially available SCHOTT leached fibers, could be significantly enhanced in the future through the development of custom-designed fibers. For example, producing leached fiber bundles with reduced inter-core spacing would improve lateral resolution and minimize transmission losses in the inter-core regions. Optimizing the manufacturing process to increase parameters such as bundle diameter, length, core density, geometry, core size, and core-to-core homogeneity will also further enhance the capabilities of the next-generation holographic fiberscopes.

RESOURCE AVAILABILITY

Lead contact

Requests for extended information and resources should be directed to the lead contact, Dr. Valentina Emiliani (valentina.emiliani@inserm.fr).

Materials availability

This study did not generate new unique reagents, mouse lines, or optical elements. Commercially available resources are indicated in the key resources table.

Data and code availability

- All data reported in this paper will be shared by the lead contact upon request.
- This paper does not report original code.
- Any additional information required to reanalyze the data reported in this paper is available from the lead contact upon request.

ACKNOWLEDGMENTS

We acknowledge support from the IHU FOReSIGHT grant (grants P-ALLOP3-IHU-000 and P-HOLO00-IHU-000), the Agence National de la Recherche (grants ANR 2MEnHoloMD – 19-CE19-0001-01 and ANR 2P-COMFIB – 23-ERCS-0009), the ERC Advanced Grant HOLOVIS (ERC-2019-AdG; award no. 885090), and the ERC Horizon 2020 H2020-ICT (DEEPER, 101016787). We would like to thank Valeria Zampini for her support in the preparation of animal protocols and Manuel Simonutti, Julie Degardin, and Pauline Abgrall from the animal facility of the Vision Institute for their help and support with animal experimentation. We thank Jana Kasik for editing the videos on DaVinci Resolve.

AUTHOR CONTRIBUTIONS

N.A., D.D., A.L.-C., F.G.C.B., and C.T. built the optical setup. D.D. developed the acquisition software and performed the optical characterization. B.F. and D.D. performed the temperature simulations. F.G.C.B. and C.T. designed and developed the implant for freely moving experiments. F.G.C.B. developed surgical procedures and performed viral injections. F.G.C.B. and M.A. performed imaging and photostimulation experiments in freely moving mice. D.D. performed the DeepLabCut analysis and the experiment on the spot propagation in scattering tissue. F.G.C.B., M.A., D.D., and N.A. analyzed the data and discussed the results with V.E. F.G.C.B., N.A., and V.E. wrote the manuscript with contributions from all authors. The manuscript was revised by V.E., F.G.C.B., and D.D., and the final version was approved by all authors. N.A. and V.E. conceived the project.

DECLARATION OF INTERESTS

The authors declare no competing interests.

STAR★METHODS

Detailed methods are provided in the online version of this paper and include the following:

- KEY RESOURCES TABLE
- EXPERIMENTAL MODEL AND STUDY PARTICIPANT DETAILS
  - Mouse lines and knockout genetics
- METHOD DETAILS
  - Imaging setup
  - Photostimulation setup
  - Fiber and GRIN lens specifications
  - Controlling software
  - Viral vectors injections and surgical procedure for *in vivo* experiments
  - Preparation of fixed mouse brain slices
  - Axial resolution measurements

- Calculation of cores illumination
- Temperature simulations
- QUANTIFICATION AND STATISTICAL ANALYSIS
  - Motion correction and movement estimation
  - Calcium data analysis
  - Tracking data processing
- ADDITIONAL RESOURCES

SUPPLEMENTAL INFORMATION

Supplemental information can be found online at https://doi.org/10.1016/j.crmeth.2026.101305.

Received: March 28, 2025
Revised: November 7, 2025
Accepted: January 7, 2026
Published: February 13, 2026

REFERENCES

1. Xu, Y., Zou, P., and Cohen, A.E. (2017). Voltage imaging with genetically encoded indicators. Curr. Opin. Chem. Biol. *39*, 1. https://doi.org/10.1016/j.cbpa.2017.04.005.
2. Lin, M.Z., and Schnitzer, M.J. (2016). Genetically encoded indicators of neuronal activity. Nat. Neurosci. *19*, 1142. https://doi.org/10.1038/nn.4359.
3. Zhang, Y., Rózsa, M., Liang, Y., Bushey, D., Wei, Z., Zheng, J., Reep, D., Broussard, G.J., Tsang, A., Tsegaye, G., et al. (2023). Fast and sensitive GCaMP calcium indicators for imaging neural populations. Nature *615*, 884. https://doi.org/10.1038/s41586-023-05828-9.
4. Emiliani, V., Entcheva, E., Hedrich, R., Hegemann, P., Konrad, K.R., Lüscher, C., Mahn, M., Pan, Z.H., Sims, R.R., Vierock, J., and Yizhar, O. (2022). Optogenetics for light control of biological systems. Nat. Rev. Methods Primers *2*, 55. https://doi.org/10.1038/s43586-022-00136-4.
5. Abdelfattah, A., Allu, S.R., Campbell, R.E., Cheng, X., Cižmár, T., Costantini, I., Emiliani, V., Fomin-Thunemann, N., Gilad, A., Fernández Alfonso, T., et al. (2022). Neurophotonic Tools for Microscopic Measurements and Manipulation: Status Report. Neurophotonics *9*, 013001. https://doi.org/10.1117/1.NPh.9.S1.013001.
6. Ronzitti, E., Ventalon, C., Canepari, M., Forget, B.C., Papagiakoumou, E., and Emiliani, V. (2017). Recent advances in patterned photostimulation for optogenetics. J. Opt. *19*, 113001. https://doi.org/10.1088/2040-8986/aa8299.
7. Aharoni, D., and Hoogland, T.M. (2019). Circuit investigations with open-source miniaturized microscopes: Past, present and future. Front. Cell. Neurosci. *13*, 141. https://doi.org/10.3389/fncel.2019.00141.
8. Scherrer, J.R., Lynch, G.F., Zhang, J.J., and Fee, M.S. (2023). An optical design enabling lightweight and large field-of-view head-mounted microscopes. Nat. Methods *20*, 546–549. https://doi.org/10.1038/s41592-023-01806-1.
9. Zong, W., Wu, R., Li, M., Hu, Y., Li, Y., Li, J., Rong, H., Wu, H., Xu, Y., Lu, Y., et al. (2017). Fast high-resolution miniature two-photon microscopy for brain imaging in freely behaving mice. Nat. Methods *14*, 713–719. https://doi.org/10.1038/nmeth.4305.
10. Bao, H., Allen, J., Pattie, R., Vance, R., and Gu, M. (2008). Fast handheld two-photon fluorescence microendoscope with a 475 μm × 475 μm field of view for in vivo imaging. Opt. Lett. *33*, 1333–1335. https://doi.org/10.1364/ol.33.001333.
11. Göbel, W., Kerr, J.N.D., Nimmerjahn, A., and Helmchen, F. (2004). Miniaturized two-photon microscope based on a flexible coherent fiber bundle and a gradient-index lens objective. Opt Lett *29*, 2521–2523. https://doi.org/10.1364/ol.29.002521.
12. Klioutchnikov, A., Wallace, D.J., Sawinski, J., Voit, K.M., Groemping, Y., and Kerr, J.N.D. (2023). A three-photon head-mounted microscope for imaging all layers of visual cortex in freely moving mice. Nat. Methods *20*, 610–616. https://doi.org/10.1038/s41592-022-01688-9.
13. Zhang, Z., Liu, S.-J., Mattison, B., Muir, J., Spurr, N., Kim, C.K., and Yang, W. (2025). High-speed neural imaging with multiplexed miniaturized two-photon microscopy. Cell Reports Methods *5*, 101221. https://doi.org/10.1016/j.crmeth.2025.101221.
14. Accanto, N., Blot, F.G.C., Lorca-Cámara, A., Zampini, V., Bui, F., Tourain, C., Badt, N., Katz, O., and Emiliani, V. (2023). A flexible two-photon fiberscope for fast activity imaging and precise optogenetic photostimulation of neurons in freely moving mice. Neuron *111*, 176. https://doi.org/10.1016/j.neuron.2022.10.030.
15. Zhang, J., Hughes, R.N., Kim, N., Fallon, I.P., Bakhurin, K., Kim, J., Severino, F.P.U., and Yin, H.H. (2023). A one-photon endoscope for simultaneous patterned optogenetic stimulation and calcium imaging in freely behaving mice. Nat. Biomed. Eng. *7*, 499–510. https://doi.org/10.1038/s41551-022-00920-3.
16. Szabo, V., Ventalon, C., De Sars, V., Bradley, J., and Emiliani, V. (2014). Spatially Selective Holographic Photoactivation and Functional Fluorescence Imaging in Freely Behaving Mice with a Fiberscope. Neuron *84*, 1157–1169. https://doi.org/10.1016/j.neuron.2014.11.005.
17. Tsvirkun, V., Sivankutty, S., Baudelle, K., Habert, R., Bouwmans, G., Vanvincq, O., Andresen, E.R., and Rigneault, H. (2019). Flexible lensless endoscope with a conformationally invariant multi-core fiber. Optica *6*, 1185. https://doi.org/10.1364/optica.6.001185.
18. Andresen, E.R., Sivankutty, S., Bouwmans, G., Gallais, L., Monneret, S., and Rigneault, H. (2015). Measurement and compensation of residual group delay in a multi-core fiber for lensless endoscopy. J. Opt. Soc. Am. B *32*, 1221. https://doi.org/10.1364/JOSAB.32.001221.
19. Garofalakis, A., Kruglik, S.G., Mansuryan, T., Gillibert, A., Thiberville, L., Louradour, F., Vever-Bizet, C., and Bourg-Heckly, G. (2019). Characterization of a multicore fiber image guide for nonlinear endoscopic imaging using two-photon fluorescence and second-harmonic generation. J. Biomed. Opt. *24*, 1–12. https://doi.org/10.1117/1.jbo.24.10.106004.
20. Ford, H.D., and Tatam, R.P. (2011). Characterization of optical fiber imaging bundles for swept-source optical coherence tomography. Appl. Opt. *50*, 627–640. https://doi.org/10.1364/AO.50.000627.
21. Stark, S.L., Gross, H., Reglinski, K., Messerschmidt, B., and Eggeling, C. (2023). Field curvature reduction in miniaturized high numerical aperture and large field-of-view objective lenses with sub 1 μm lateral resolution. Biomed. Opt. Express *14*, 6190–6205. https://doi.org/10.1364/boe.499785.
22. Jung, G.S., and Won, Y.H. (2020). Simple and fast field curvature measurement by depth from defocus using electrowetting liquid lens. Appl. Opt. *59*, 5527. https://doi.org/10.1364/ao.394565.
23. Zong, W., Obenhaus, H.A., Skytøen, E.R., Eneqvist, H., de Jong, N.L., Vale, R., Jorge, M.R., Moser, M.B., and Moser, E.I. (2022). Large-scale two-photon calcium imaging in freely moving mice. Cell *185*, 1240. https://doi.org/10.1016/j.cell.2022.02.017.
24. Parker, H.E., Perperidis, A., Stone, J.M., Dhaliwal, K., and Tanner, M.G. (2020). Core crosstalk in ordered imaging fiber bundles. Opt. Lett. *45*, 6490–6493. https://doi.org/10.1364/ol.405764.
25. Helmchen, F., Tank, D.W., and Denk, W. (2002). Enhanced two-photon excitation through optical fiber by single-mode propagation in a large core. Appl. Opt. *41*, 2930–2934. https://doi.org/10.1364/ao.41.002930.
26. Picot, A., Dominguez, S., Liu, C., Chen, I.-W., Tanese, D., Ronzitti, E., Berto, P., Papagiakoumou, E., Oron, D., Tessier, G., et al. (2018). Temperature Rise under Two-Photon Optogenetic Brain Stimulation. Cell Rep. *24*, 1243–1253.e5. https://doi.org/10.1016/j.celrep.2018.06.119.
27. Podgorski, K., and Ranganathan, G. (2016). Brain heating induced by near-infrared lasers during multiphoton microscopy. J. Neurophysiol. *116*, 1012–1023. https://doi.org/10.1152/jn.00275.2016.

28. Reig, R., Mattia, M., Compte, A., Belmonte, C., and Sanchez-Vives, M.V. (2010). Temperature Modulation of Slow and Fast Cortical Rhythms. J. Neurophysiol. *103*, 1253–1261. https://doi.org/10.1152/jn.00890.2009.

29. Andersen, P., and Moser, E.I. (1995). Brain temperature and hippocampal function. Hippocampus *5*, 491–498. https://doi.org/10.1002/hipo.450050602.

30. Thompson, S.M., Masukawa, L.M., and Prince, D.A. (1985). Temperature dependence of intrinsic membrane properties and synaptic potentials in hippocampal CA1 neurons in vitro. J. Neurosci. *5*, 817–824. https://doi.org/10.1523/JNEUROSCI.05-03-00817.1985.

31. Thomsen, S. (1991). PATHOLOGIC ANALYSIS OF PHOTOTHERMAL AND PHOTOMECHANICAL EFFECTS OF LASER–TISSUE INTERACTIONS. Photochem. Photobiol. *53*, 825–835. https://doi.org/10.1111/j.1751-1097.1991.tb09897.x.

32. Papagiakoumou, E., Bègue, A., Leshem, B., Schwartz, O., Stell, B.M., Bradley, J., Oron, D., and Emiliani, V. (2013). Functional patterned multiphoton excitation deep inside scattering tissue. Nat. Photonics *7*, 274–278. https://doi.org/10.1038/nphoton.2013.9.

33. Bègue, A., Papagiakoumou, E., Leshem, B., Conti, R., Enke, L., Oron, D., and Emiliani, V. (2013). Two-photon excitation in scattering media by spatiotemporally shaped beams and their application in optogenetic stimulation. Biomed. Opt. Express *4*, 2869–2879. https://doi.org/10.1364/BOE.4.002869.

34. Liu, Y., Zhang, J., Lee, C.Y., Zhang, H., and Li, X. (2025). Alleviating head-mounted weight burden for neural imaging in freely-behaving rodents. Sci. Rep. *15*, 19175. https://doi.org/10.1038/s41598-025-04300-0.

35. Giovannucci, A., Friedrich, J., Gunn, P., Kalfon, J., Brown, B.L., Koay, S.A., Taxidis, J., Najafi, F., Gauthier, J.L., Zhou, P., et al. (2019). Caiman an open source tool for scalable calcium imaging data analysis. eLife *8*, e38173. https://doi.org/10.7554/eLife.38173.

36. Ramirez, J.E., and Stell, B.M. (2016). Calcium Imaging Reveals Coordinated Simple Spike Pauses in Populations of Cerebellar Purkinje Cells. Cell Rep. *17*, 3125–3132. https://doi.org/10.1016/j.celrep.2016.11.075.

37. Roome, C.J., and Kuhn, B. (2018). Simultaneous dendritic voltage and calcium imaging and somatic recording from Purkinje neurons in awake mice. Nat. Commun. *9*, 3388. https://doi.org/10.1038/s41467-018-05900-3.

38. Lutz, C., Otis, T.S., DeSars, V., Charpak, S., DiGregorio, D.A., and Emiliani, V. (2008). Holographic photolysis of caged neurotransmitters. Nat. Methods *5*, 821–827. https://doi.org/10.1038/nmeth.1241.

39. Papagiakoumou, E., de Sars, V., Oron, D., and Emiliani, V. (2008). Patterned two-photon illumination by spatiotemporal shaping of ultrashort pulses. Opt. Express *16*, 22039–22047. https://doi.org/10.1364/oe.16.022039.

40. Yeminy, T., and Katz, O. (2021). Guidestar-free image-guided wavefront shaping. Sci. Adv. *7*, eabf5364. https://doi.org/10.1126/sciadv.abf5364.

41. Perkins, L.N., Semu, D., Shen, J., Boas, D.A., and Gardner, T.J. (2018). High-density microfibers as a potential optical interface to reach deep brain regions. J. Neural. Eng. *15*, 066002. https://doi.org/10.1088/1741-2552/aadbb2.

42. Lorca-Cámara, A., Tourain, C., de Sars, V., Emiliani, V., and Accanto, N. (2024). Multicolor two-photon light-patterning microscope exploiting the spatio-temporal properties of a fiber bundle. Biomed. Opt. Express *15*, 2094. https://doi.org/10.1364/boe.507690.

43. Futia, G.L., Zohrabi, M., McCullough, C., Teel, A., Simoes de Souza, F., Oroke, R., Miscles, E.J., Ozbay, B.N., Kilborn, K., Bright, V.M., et al. (2024). Opto2P-FCM: A MEMS based miniature two-photon microscope with two-photon patterned optogenetic stimulation. Preprint at bioRxiv. https://doi.org/10.1101/2024.10.21.619528.

44. Klioutchnikov, A., and Kerr, J.N.D. (2023). Chasing cortical behavior: designing multiphoton microscopes for imaging neuronal populations in freely moving rodents. Neurophotonics *10*, 044411. https://doi.org/10.1117/1.NPh.10.4.044411.

45. Carrillo-Reid, L., Han, S., Yang, W., Akrouh, A., and Yuste, R. (2019). Controlling Visually Guided Behavior by Holographic Recalling of Cortical Ensembles. Cell *178*, 447–457.e5. https://doi.org/10.1016/j.cell.2019.05.045.

46. Dalgleish, H.W., Russell, L.E., Packer, A.M., Roth, A., Gauld, O.M., Greenstreet, F., Thompson, E.J., and Häusser, M. (2020). How many neurons are sufficient for perception of cortical activity? eLife *9*, e58889. https://doi.org/10.7554/eLife.58889.

47. Shin, H., Ogando, M.B., Abdeladim, L., Jagadisan, U.K., Durand, S., Hardcastle, B., Belski, H., Cabasco, H., Loefler, H., Bawany, A., et al. (2025). Recurrent pattern completion drives the neocortical representation of sensory inference. Nat. Neurosci. *28*, 2319–2329. https://doi.org/10.1038/s41593-025-02055-5.

48. Sridharan, S., Gajowa, M.A., Ogando, M.B., Jagadisan, U.K., Abdeladim, L., Sadahiro, M., Bounds, H.A., Hendricks, W.D., Turney, T.S., Tayler, I., et al. (2022). High-performance microbial opsins for spatially and temporally precise perturbations of large neuronal networks. Neuron *110*, 1139. https://doi.org/10.1016/j.neuron.2022.01.008.

49. Kishi, K.E., Kim, Y.S., Fukuda, M., Inoue, M., Kusakizako, T., Wang, P.Y., Ramakrishnan, C., Byrne, E.F.X., Thadhani, E., Paggi, J.M., et al. (2022). Structural basis for channel conduction in the pump-like channelrhodopsin ChRmine. Cell *185*, 672. https://doi.org/10.1016/j.cell.2022.01.007.

50. Aggarwal, A., Sunil, S., Bendifallah, I., Moon, M., Drobizhev, M., Zarowny, L., Zheng, J., Wu, S.-Y., Lohman, A.W., Tebo, A.G., et al. (2024). Blue-shifted genetically encoded Ca2+ indicator with enhanced two-photon absorption. Neurophotonics *11*, 024207. https://doi.org/10.1117/1.NPh.11.2.024207.

51. Lorca-Cámara, A., Blot, F.G.C., and Accanto, N. (2024). Recent advances in light patterned optogenetic photostimulation in freely moving mice. Neurophotonics *11*, S11508. https://doi.org/10.1117/1.nph.11.s1.s11508.

52. Zong, W., Wu, R., Chen, S., Wu, J., Wang, H., Zhao, Z., Chen, G., Tu, R., Wu, D., Hu, Y., et al. (2021). Miniature two-photon microscopy for enlarged field-of-view, multi-plane and long-term brain imaging. Nat. Methods *18*, 46–49. https://doi.org/10.1038/s41592-020-01024-z.

53. Händel, B.F., and Schölvinck, M.L. (2019). The brain during free movement – What can we learn from the animal model. Brain Res. *1716*, 3. https://doi.org/10.1016/j.brainres.2017.09.003.

54. Lyu, C., Yu, C., Sun, G., Zhao, Y., Cai, R., Sun, H., Wang, X., Jia, G., Fan, L., Chen, X., et al. (2022). Deconstruction of Vermal Cerebellum in Ramp Locomotion in Mice. Adv. Sci. *10*, e2203665. https://doi.org/10.1002/advs.202203665.

55. Aghajan, Z.M., Acharya, L., Moore, J.J., Cushman, J.D., Vuong, C., and Mehta, M.R. (2015). Impaired spatial selectivity and intact phase precession in two-dimensional virtual reality. Nat. Neurosci. *18*, 121–128. https://doi.org/10.1038/nn.3884.

56. Rector, D., and Harper, R. (1991). Imaging of hippocampal neural activity in freely behaving animals. Behav. Brain Res. *42*, 143–149. https://doi.org/10.1016/S0166-4328(05)80005-1.

57. Bounds, H.A., Sadahiro, M., Hendricks, W.D., Gajowa, M., Gopakumar, K., Quintana, D., Tasic, B., Daigle, T.L., Zeng, H., Oldenburg, I.A., and Adesnik, H. (2023). All-optical recreation of naturalistic neural activity with a multifunctional transgenic reporter mouse. Cell Rep. *42*, 112909. https://doi.org/10.1016/j.celrep.2023.112909.

58. Drinnenberg, A., Attinger, A., Raventos, A., Ramakrishnan, C., Gharagoz, B.E., Cordero, A., Jo, Y., Siverts, L., Daigle, T.L., Tasic, B., et al. (2025). Large-scale cellular-resolution read/write of activity enables discovery of cell types defined by complex circuit properties. Preprint at bioRxiv. https://doi.org/10.1101/2025.10.21.683734.

59. Mathis, A., Mamidanna, P., Cury, K.M., Abe, T., Murthy, V.N., Mathis, M.W., and Bethge, M. (2018). DeepLabCut: markerless pose estimation of user-defined body parts with deep learning. Nat. Neurosci. *21*, 1281–1289. https://doi.org/10.1038/s41593-018-0209-y.

60. Gerchberg, R.W., and Saxton, W.O. (1972). Practical algorithm for the determination of phase from image and diffraction plane pictures. Optik *35*, 237.

61. Li, C.X., Patel, S., Auerbach, E.J., and Zhang, X. (2013). Dose-dependent effect of isoflurane on regional cerebral blood flow in anesthetized macaque monkeys. Neurosci. Lett. *541*, 58–62. https://doi.org/10.1016/j.neulet.2013.02.007.

62. Chang, L.C., Raty, S.R., Ortiz, J., Bailard, N.S., and Mathew, S.J. (2013). The Emerging Use of Ketamine for Anesthesia and Sedation in Traumatic Brain Injuries. CNS Neurosci. Ther. *19*, 390. https://doi.org/10.1111/cns.12077.

63. Carslaw, H.S.&J.J.C. (1959). Conduction of Heat in Solids (Clarendon Press).

64. Cheng, X., Li, Y., Mertz, J., Sakadžić, S., Devor, A., Boas, D.A., and Tian, L. (2019). Development of a beam propagation method to simulate the point spread function degradation in scattering media. Opt. Lett. *44*, 4989–4992. https://doi.org/10.1364/OL.44.004989.

CellPress
OPEN ACCESS

STAR★METHODS

KEY RESOURCES TABLE

REAGENT or RESOURCE	SOURCE	IDENTIFIER
**Bacterial and virus strains**		
AAV9-*syn*-jGCaMP7s-WPRE	Addgene	Cat#104487-AAV9; RRID: Addgene_104487
AAV1-hSyn-ChRmine-mscarlet-Kv2.1-WPRE	Vision Institute vector core facility - Plasmid Addgene	Cat#130995; RRID:Addgene_130995
**Chemicals, peptides, and recombinant proteins**		
Dexazone (Dexamethasone) - Virbac	Centravet	Cat#DEX216
Laocaine (Lidocaine) - MSD	Centravet	Cat#LAO001
Rompun 2% (Xylazine) - Bayer	Centravet	Cat#ROM001
Ketamidor (Ketamine) - Axience	Axience Santé Animale	Cat#152500
Lubrithal (Eye gel) - Dechra	Centravet	Cat#LUB001
Buprenorphine	Axience Santé Animale	Cat#151244
Isoflurane	Axience Santé Animale	Cat#153613
Antisedan-ANTIDORM	Axience Santé Animale	Cat#152494
Tetric Evoflow A1-IVOCLAR	Dentaltix	Ca#45TE595953
**Experimental models: Organisms/strains**		
C57BL/6J	Janvier Labs	https://janvier-labs.com/fiche_produit/2-c57bl-6jrj/; RRID:IMSR_RJ:C57BL-6NRJ
**Software and algorithms**		
WaveFront V	Emiliani's Lab	Available from: vincent.de-sars@inserm.fr
FIJI/ImajeJ 2.0.0	ImageJ	https://imagej.net/software/fiji/; RRID:SCR_002285
LabView 2016	National Instruments	https://www.ni.com/fr-fr/shop/labview.html; RRID:SCR_014325
DeepLabCut 2.3.9	Mathis's Lab (Mathis et al.[59])	https://github.com/DeepLabCut/DeepLabCut; RRID:SCR_021391
CaImAn 1.9.7	Flatiron Institute	https://github.com/flatironinstitute/CaImAn; RRID:SCR_021533
**Other**		
Digilent Discovery 3	Digilent	https://digilent.com/reference/test-and-measurement/analog-discovery-3/start
Digilent Discovery 2	Digilent	https://digilent.com/reference/test-and-measurement/analog-discovery-2/start
EM-CCD Camera iXon Ultra 888	Andor - Oxford Instruments	https://andor.oxinst.com/products/ixon-emccd-cameras-for-physical-science; RRID:SCR_023166
Laser NKT Photonics – AEROPULSE FS50	NKT Photonics	https://www.nktphotonics.com/products/ultrafast-fiber-lasers/aeropulse-fs-50/
LCOS-SLM X13138-07	Hamamatsu	https://www.hamamatsu.com/eu/en/product/optical-components/lcos-slm.html; RRID:SCR_017105
"BundleF" Fujikura Fiber FIGH-15-600N	Fujikura	https://www.optic-product.fujikura.com/optical-fibers/en/products/image-fiberfigh-series-n-type/
"S-BundleS" SCHOTT Fiber IB1010895CTD	Micro Optics Europe	Cat#3049
"L-BundleS" SCHOTT Fiber IB1651350	Micro Optics Europe	Cat#3219
GRIN Lens: GT-MO-080-032-ACR-VISNIR-08CG-20	Grintech	https://www.grintech.de/en/products/miniature-high-na-lenses/

(*Continued on next page*)

Continued

REAGENT or RESOURCE	SOURCE	IDENTIFIER
Mini-objective D0213-3X (D3X-5W1)	DomiLight/Thorlabs	https://www.thorlabs.com/thorproduct.cfm?partnumber=D3X-5W1
Fiber bundle holder (custom made)	Emiliani's lab	https://github.com/photonics-VisionInstitute/2pFendo
Mouse head implant (3D printed, custom made)	Emiliani's lab	https://github.com/photonics-VisionInstitute/2pFendo
IR mirrors BB2-E03	Thorlabs	https://www.thorlabs.com/thorproduct.cfm?partnumber=BB2-E03
IR Achromatic doublets (lenses)	Thorlabs	https://www.thorlabs.com/newgrouppage9.cfm?objectgroup_id=259
Short-pass 1000nm cutoff dichroic (laser coupling)	Thorlabs	https://www.thorlabs.com/thorproduct.cfm?partnumber=DMSP1000R
Long-pass 650nm dichroic (fluo collection)	Thorlabs	https://www.thorlabs.com/thorproduct.cfm?partnumber=DMLP650R
IR blocking TF1 filter	Thorlabs	https://www.thorlabs.com/thorproduct.cfm?partnumber=TF1
Mitutoyo Plan, MY10X-803	Thorlabs	https://www.thorlabs.com/thorproduct.cfm?partnumber=MY10X-803
Fluorescence filter BrightLine 550/88nm	Semrock	https://www.idex-hs.com/store/product-detail/ff01_550_88_25/fl-003868
100mm Tube Lens TTL100-A	Thorlabs	https://www.thorlabs.com/thorproduct.cfm?partnumber=TTL100-A
Polarizing beam splitter (920nm laser)	Thorlabs	https://www.thorlabs.com/thorproduct.cfm?partnumber=PBS125
λ/2 plate (920nm laser)	Thorlabs	https://www.thorlabs.com/thorproduct.cfm?partnumber=WPH10M-915
λ/2 plate (1030nm laser)	Thorlabs	https://www.thorlabs.com/thorproduct.cfm?partnumber=WPH10M-1030
Rotating motor & controller	Thorlabs	https://www.thorlabs.com/newgrouppage9.cfm?objectgroup_id=2875
Shutter (SH05R)	Thorlabs	https://www.thorlabs.com/thorproduct.cfm?partnumber=SH05R/M
Shutter controller (KSC101)	Thorlabs	https://www.thorlabs.com/thorproduct.cfm?partnumber=KSC101
Z-motor (Ezi motion plus r v6)	Fastech	https://fastech.co.kr/new/eng/main.php

EXPERIMENTAL MODEL AND STUDY PARTICIPANT DETAILS

Mouse lines and knockout genetics

Wild-type males and females C57BL/6J mice (Janvier Labs) were used for experiments (p80-110 for injection and p110-140 for imaging, p stands for "postnatal day"). All experiments were performed in accordance with EU Directive 2010/63. The protocols were reviewed by the local animal experimentation ethics committee (CETEA n.44) and authorized by the French Ministry of Research and Education (#201803261541580). Advice on procedures, refinement of animal experimentation standards, and pain and distress management are provided by the Local Animal Welfare Office. Animals are housed in 2–5 per cage, with a light-dark cycle of 12 + 12 h, and food and water *ad libitum*.

METHOD DETAILS

Imaging setup

The imaging laser was an ultrafast fiber laser (Spark Alcor, central wavelength of 920 nm, pulse duration of 130 fs, total power of 4 W, repetition rate of 80 MHz), whose output power was controlled by using a lambda half waveplate and a polarizing beam splitter. The internal laser compressor was set to ~ - 90000 fs^2 to compensate for the fiber dispersion. The laser beam was first magnified using two lenses of 100 and 200mm for 2X magnification. It was then sent to the scanning galvanometric mirrors placed in a Fourier plane of the fiber entrance, passed through one lens of 200mm to focus the laser beam on a circular iris plane also placed in the Fourier plane

of the mirrors used to fit the circular aperture of the fiber. This cropping primarily prevents the scanning laser from reaching the external metallic coating of the fiber entrance, which could cause internal reflections within the system. Additionally, it removes the lateral edges of the scanning area, where the horizontal scanning mirror reverses direction. This reversal leads to longer exposure times and, consequently, inhomogeneous illumination.

The cropped laser scan passed through a 400mm lens for 2X magnification. A 200mm lens was then used to couple the scanned laser beam into the fiber. The average power measured after the iris was ~65% of the power measured before. It was improved compared to the previous system (~50%) by changing the scanning method from a cosinus function to a triangular function, leading to shorter scanning time at the edges where the mirrors decrease their speeds to reverse the direction. The emission from the sample was collected through the fiber and filtered with a long pass dichroic beam splitter to separate it from the incoming laser beam. With a theoretical 10X microscope objective (Mitutoyo Plan, MY10X-803) and a tube-lens of 100mm (Thorlabs TTL100-A) resulting in a practical 5X magnification we could image onto the EM-CCD camera (Andor Ultra 888, 1024 × 1024 pixels, pixel size 13.3 × 13.3 μm^2). Between the objective and the tube-lens, two IR blocking filters (Thorlabs TF1) and a green fluorescence emission filter (BrightLine 550/88nm) were placed. The camera was used in crop mode with a sub array of 600 × 600 pixels.

Photostimulation setup

The photostimulation laser was a low repetition rate fiber laser (NKT, central wavelength of 1030 nm, pulse duration of 420 fs, total power of 54 W, repetition rate of 1.2 MHz) whose power was internally controlled. The laser beam was magnified by two lenses of 100 and 500 mm (5X) and sent to an LC-SLM (LCOS-SLM X13138-07, Hamamatsu Photonics, resolution 1272 × 1024 pixels, 12.5 μm pixel size) to be spatially shaped. Spatial shaping is achieved by controlling the LC-SLM with holographic phase profiles generated by the software WavefrontV using a Gerchberg-Saxton phase retrieva algorithm.[60] Two lenses of 500 and 750 mm conjugated the LC-SLM plane with the back focal plane of a last 150 mm coupling lens. A short pass dichroic mirror was used to recombine the photostimulation and imaging beams before being focused into the fiber proximal end. The laser beam was made slightly divergent with the first 5X telescope before arriving onto the LC-SLM, which applied a defocus to the beam to compensate for the divergence. In this way we separated in z the planes of the 0th and 1st diffraction order of the LC-SLM. A small beam blocker was then used to block the 0th order without affecting the central part of the holographic FOV.

Fiber and GRIN lens specifications

The fiber bundles used were a 2 m Fujikura model FIGH-15-600N (BundleF), a 0.895 m SCHOTT model IB1010895CTD (S-BundleS) and a 1.35 m model IB1651350 (L-BundleS) which were coupled to either chromatic and field corrected GRIN lens Grintech models GT-MO-080-032-ACR-VISNIR-08CG-20, diameter of 1.3 mm, or a mini-objective DomiLight D0213-3X, diameter of 3mm. The chromatic correction allows both the fluorescence and two laser wavelengths, for imaging and photostimulation, to be focused on the same plane. Fiber bundles and GRIN lens or mini-objective were held together by a custom-made 3D printed casing in resin adapted from Zong et al.[23]

Controlling software

A custom-made software written in LabVIEW was used to control the shutter for the imaging laser, the lambda half waveplate for control of the imaging power, the frequency and amplitude of scanning for the galvanometric mirrors, the power and internal shutter of the photostimulation laser, the z step motor used to move vertically the fiberscope, and the XY position stage. The software also controlled the frequency, crop and gain of the EM-CCD camera (Andor Solis) and synchronized it with the z step motor for z stack and the internal shutter of the 1030nm laser for photostimulation experiments. During photostimulation experiments the internal shutter of the photostimulation laser opened for 20 ms every 250 ms. The EM-CCD camera was turned off for 50 ms in correspondence to the periods of photostimulation illumination to remove completely any artifact that comes from the photostimulation laser and resulting in a 4Hz acquisition.

A custom-designed software, Wavefront-Designer V, written in C++ and using the open framework Qt 5.15.2, calculated the phase profile and controlled the LC-SLM for the dynamic configuration of the hologram, using Gerchberg–Saxton-based algorithms. The software also included an optical aberration correction based on Zernike polynom.

Viral vectors injections and surgical procedure for *in vivo* experiments

Viral injections and cranial window were performed during a single surgical procedure (~1h10) over either a cortical region (V1 or wS1) or the cerebellum (CbC). All surgical procedures were performed with a mixed anesthesia based on intraperitoneal injection of ketamine (60–80 mg/Kg) - xylazine (5–8 mg/Kg) for the first 40–45 min, followed by isoflurane inhalation in air (<0.75%) for <30min. The use of mixed anesthesia is due to the different properties of anesthetics. Isoflurane, as an inhaled anesthetic, is associated with a decrease in cerebral vascular resistance and induces vasodilatation, which increases cerebral blood flow and intracranial pressure.[61] This is particularly evident when the dura mater is open, promoting bleeding and brain herniation. In contrast, ketamine, although it can temporarily increase blood pressure and heart rate, generally does not have a significant impact on intracranial pressure and even helps to maintain intracranial pressure due to its sympathetic stimulation effects.[62] However, ketamine effect wears off in 40-45min and maintenance with re-injection of ketamine is deleterious for the animal's recovery. The control of intracranial pressure is essential as brain herniation results, after sealing the window and re-equilibration of intracranial pressure, in a marked space

between brain surface and cover-glass prompt for fibrotic tissue growth. The anti-inflammatory dexamethasone sodium phosphate (0.01 mg/g) was injected subcutaneously 16h and 2h prior to the surgery, and analgesic buprenorphine (0.1mg/Kg) > 40min before. Lidocaine 2% was injected subcutaneously before opening the skin. Eyes were protected with ophthalmic gel, and the body temperature was kept constant at 37°C (homeothermic system, Kent Scientific). Subcutaneous injections of 0.1 mL sterile saline solution ensured rehydration all through the experiments.

A cranial window of 3–3.5 mm was made, the dura was removed while keeping the brain surface moist with sterile saline solution. Viruses were delivered at high titer of adeno-associated viral vectors (viral concentration in the order of 10^{12} vp/ml) for expression of jGCaMP7s alone or jGCaMP7s together with the opsin ChRmine. The following viral constructs were used: AAV9-*syn*-jGCaMP7s-WPRE (Addgene) or a mixture 2:1 of AAV9-*syn*-jGCaMP7s-WPRE + AAV1-hSyn-ChRmine-mscarlet-Kv2.1-WPRE. Four injections of 150-200nL of viral solution were done within the window, at ~200μm depth from the brain surface. A 3 mm cover glass (0.1mm thickness; Multichannel System) was placed on top of the craniotomy and sealed with dental cement (Tetric EvoFlow A2, Ivoclar). The head plate was then fixed with dental cement (Super-Bond Universal Kit, SUN MEDICAL). For blood vessels visualization (Figure S3A) only the cranial window was made during the surgical procedure. On the experimental day, the animal was head fixed and anesthetized, and a retro-orbital injection of fluorescin was performed for subsequent acquisitions in the following hour.

One month after the surgical procedure, the animals were habituated to awake head fixation on a treadmill (Janelia 2017-049, https://doi.org/10.25378/janelia.24691311) before the first imaging sessions. The fully assembled 2P-FENDO-II (fiber + casing + mini-objective + base plate) was hold by a motorized z-stage while the treadmill rests on a x/y motorized stage. The water immersion normally used for the mini-objective was replaced with an ultrasound gel of similar refractive index and absorption to prevent leakage and/or evaporation over the course of the experiment. This did not affect signal intensity, image quality or the focal plane position. On the first day of experiments the animal was head fixed bellow the 2P-FENDO-II on its holder in order to navigate it above the cranial window to search for a region of interest in x/y/z. The motorized z-stage (Thorlabs DDS100) allowed, before definitive fixation of the base plate, to parameter a z stack over a region of interest (Figure S1). Upon selection of an optimal x/y/z region, the base plate was cemented (Super-Bond Universal Kit, SUN MEDICAL) to hold the system in its definitive position. The complete apparatus was then detached from its motorized holder to rest solely on the mouse head. On the following experimental days, only few minutes are then needed to clip-in the casing with the fiber and mini-objective while the animal is head fixated awake on the treadmill before being released (Figure 3C.). During freely moving experiments, the 1.35m fiber was attached to helium balloons to prevent tension that could damage the fiber while allowing the animal full freedom of movement (Figure 2A.). Animals were recorded in their home cage with a GoPro© fixed above it.

Preparation of fixed mouse brain slices

Wild-type C57BL/6J brain tissue was collected using either immersion fixation or transcardiac perfusion. For **immersion fixation**, mice were sacrificed, and whole brains were carefully extracted and post-fixed in 4% paraformaldehyde (PFA) at 4°C overnight. Following fixation, brains were washed three times with phosphate-buffered saline (PBS) and coronally sectioned in cold PBS using a Leica VT1100S vibratome equipped with razor blades. Sections of 100, 150, and 200 μm thickness were collected, transferred into PBS, and stored at 4°C until further use. For **transcardiac perfusion**, animals were deeply anesthetized by intraperitoneal injection of a ketamine (60–80 mg/kg) and xylazine (5–8 mg/kg) mixture, followed immediately by transcardial perfusion with saline and then 4% PFA in 0.12 M phosphate buffer (PB; pH 7.6). Brains were not post-fixed but were transferred directly to a 30% sucrose/PB solution overnight at 4°C. Sagittal sections were cut using a freezing microtome. Free-floating sections were then rinsed in 0.1 M PB.

Axial resolution measurements

For characterizing the axial resolution and performances of the 2P-FENDO-II, both for the imaging and photostimulation lasers, a thin (~1 μm) spin-coated fluorescent layer of rhodamine-6G in polymethyl methacrylate 2% w/v in chloroform was illuminated through the endoscope with a Gaussian spot of 10 μm FWHM and the generated 2P fluorescence was collected back through the fiber and imaged to the camera detector. By using a z-step motor (FASTECH Ezi motion plus r v6), the endoscope (fiber+ GRIN lens or mini-objective) was scanned over the desired *z* range to collect 3D stacks of images. We analyzed the recorded stacks using Python and ImageJ. For each plane in the stack, the 2P fluorescence values were determined by integrating the intensity of all pixels within a circular area containing the (imaging or photostimulation) spot. The reported axial confinement values correspond to the FWHM of the resulting curves.

To evaluate the axial broadening as a function of penetration depth, we repeated the experiment by placing fixed brain slices of increasing thickness (100, 140, 150, 160 and 200μm) on top of the rhodamine layer. From the dependence of fluorescence intensity on slice thickness, we extracted the corresponding attenuation length according to the expression $S = S_0 e^{-2z/l_e}$. Depending on the different preparations and the position of the spot within the slices, the attenuation length ranged from 80μm to 246μm. To account for this variability, we plotted the axial broadening as a function of the penetration depth normalized to the corresponding attenuation length.

Calculation of cores illumination

In this manuscript, we have defined the diameter of a photostimulation or imaging spot as the full width at half maximum (FWHM) of the 2P excitation profile at the sample plane, as recorded by the camera; this value is denoted here $FWHM_{2P}$.

CellPress
OPEN ACCESS

To estimate the average laser power delivered per fiber core (P_{core}), we divide the total laser power of a static beam P_{tot} (measured at the fiber output) by the number of illuminated cores N_{cores}. The calculation proceeds as follows:

First, we infer the FWHM of the Gaussian beam intensity, which is larger than the FWHM of the 2P intensity by a factor of $\sqrt{2}$. We then divide the square of this value by the square of the effective inter-core distance at the sample plane, which serves as our estimate for N_{cores}. The effective inter-core distance is obtained by dividing the inter-core spacing of the fiber bundle by the magnification of the micro-objective located after the fiber. In the present setup, this yields $\Delta x = 3.9\ \mu m$. Thus:

$$N_{cores} = \frac{\left(FWHM_{2P} \times \sqrt{2}\right)^2}{\Delta x^2}$$

For example, a 10 μm spot ($FWHM_{2P}$) corresponds to approximately 13 illuminated cores.

Next, to calculate P_{core}, we consider that in a Gaussian beam, 50% of the total measured power is contained within its FWHM. Therefore:

$$P_{core} = \frac{P_{tot} * 0.5}{N_{cores}}$$

Applying this to the example above, for a 10 μm spot and $P_{tot} = 200$ mW, the estimated average power per core is $P_{core} = 7.8$ mW.

Temperature simulations

The heat diffusion and temperature rise simulations were done using the Green's function formalism for a homogeneous and isotropic (infinite) medium model.[26,63] The heat distribution was obtained by calculating the propagation of the field throughout the medium. In all cases, the depth was set to 120μm below the brain surface and the absorption above the surface at 920nm was not considered. The value of simulated temperature rise was taken as the integral of the temperature value at the central pixel at x,y,z = 0,0,0. It should be noted that ΔT calculated here is the increase to the steady-state temperature in the brain. Scattering was modeled using the beam propagation method which has been shown to be particularly effective for modeling ligh propagation in biological media, where scattering is predominantly forward. This relies on successive random phase masks, whose statistical properties can be related to the macroscopic scattering properties.[64] For the imaging simulations, the heat source corresponds to the successive position of the laser beam within the FOV during the scanning. The simulations were limited to 16 consecutive frames as the equilibrium state was reached (see Figure 1) for each acquisition speed.

QUANTIFICATION AND STATISTICAL ANALYSIS

Motion correction and movement estimation

We used CaImAn[35] for motion correction, component selection, traces extraction and signal analysis. We used non-rigid motion correction with 20 × 20 μm patches. For each frame *f* in the acquisition and for each patch *p*, we obtained the *x* and *y* displacement of the FOV in μm, called dx(f,p) and dy(f,p), from which we calculated the total displacement, according to $d(f,p) = dy(f,p)^2 + dy(f,p)^2$. We then averaged d(f,p) over all patches to obtain a mean displacement for each frame, i.e., ⟨d(f)⟩p. We did not consider patches at the corners of the images as they contained a significant number of pixels that corresponded to areas outside of the fiber bundle and therefore would remain static during acquisition.

Calcium data analysis

After motion correction, we let CaImAn automatically detect active neurons. We confirmed that the detected regions of interest (ROIs) corresponded to actual cells by visually inspecting the images and manually removing ROIs that did not match neurons. For each found neuron, we used the SNR value produced by CaImAn, according to the definition given in ref.[35] To calculate DF/F traces, we used the *detrend_df_f* function from CaImAn.

The algorithm was run with the following parameters, following the CaImAn conventions: *gnb* = 2 (global background components); low thresholds: SNR_lowest = 0.5, cnn_lowest = 0.1; high thresholds: rval_thr = 0.7; min_SNR = 2; cnn_thr = 0.7. A neuron has to exceed all the low thresholds and at least one high threshold to be accepted. Occasionally, after manual inspection we added few cells with SNR<0.5. We briefly remind that *rval* measures the spatial footprint consistency of each found component; *SNR* is calculated from strong peaks of fluorescence in a trace and considering the baseline noise over the whole trace; the *cnn* based classification quantifies the resemblance of a component to a neuronal soma using a 4-layer convolutional neuronal network.

For photostimulation data we always repeated the same photostimulation patterns four consecutive times. We then averaged the DF/F signal over the four consecutive repetitions, generating the average <DF/F >. We used this average signal to determine if a neuron was or not photostimulated and to calculate the induced increase of fluorescence signal $\langle DF/F \rangle_{Photostim} = \langle DF/F \rangle_{After} - \langle DF/F \rangle_{Before}$ in response to photostimulation. We compare the average signal 2s before photostimulation $\langle DF/F \rangle_{Before}$ (which constitutes our baseline signal) with the average signal during the 2s following the middle of the photostimulation period $\langle DF/F \rangle_{After}$. As the photostimulation was composed by 10 pulses, we calculated $\langle DF/F \rangle_{After}$ by averaging over 2s after the 5th photostimulation pulse. A cell (either targeted or non-targeted) was considered photostimulated if $\langle DF/F \rangle_{Photostim}$ was greater than 3 times the

standard deviation of the baseline signal. We then plotted the probability for a cell to be photostimulated relative to the distance from a photostimulated cell and fitted the data with a single exponential decay function A*exp(−x/k), with x vector of distances from the photostimulated cells, A normalization factor, k = 18.4 ± 0.3 μm characteristic decay distance.

Tracking data processing

The tracking videos were first analyzed in DeepLabCut, trained to recognize the back of the freely moving mice with 40 labeled datasets from different mice videos, with different tracking camera mice cage positions and illumination conditions. From DeepLabCut we extracted the back part positions in pixels for each frame. Data were manually inspected to confirm position detection and to remove pose estimation errors coming from possible reflections from the sides of the cages. To calculate the velocity of the mice we divided the distance of the back between two adjacent frames by the time interval between these two frames. We then smoothed the velocity calculation over time bins of 200ms to avoid unrealistic speed artifacts. To plot the probability distribution of the mouse speed, we used for each animal 4 different videos of the animal freely-moving with and without the fiber during different experiments.

In order to quantify the mouse exploration, we divided the cage in 48 zones of 4.625 × 3.5cm and then divided the number of zones visited over the total number of zones. The exploration rate was then compared when the mouse was wearing the 2P-FENDO-II or not.

ADDITIONAL RESOURCES

Future updated versions of the system, 3D models and drawings of custom components can be found here: https://github.com/photonics-VisionInstitute/2pFendo.

Cell Reports Methods, Volume *6*

Supplemental information

2P-FENDO-II: A fiber bundle microscope for all-optical, large field-of-view brain studies in freely moving mice

François G.C. Blot, Dimitri Decombe, Antonio Lorca-Cámara, Maya Anquetil, Vincent de Sars, Christophe Tourain, Benoît C. Forget, Nicolò Accanto, and Valentina Emiliani

SUPPLEMENTAL INFORMATION

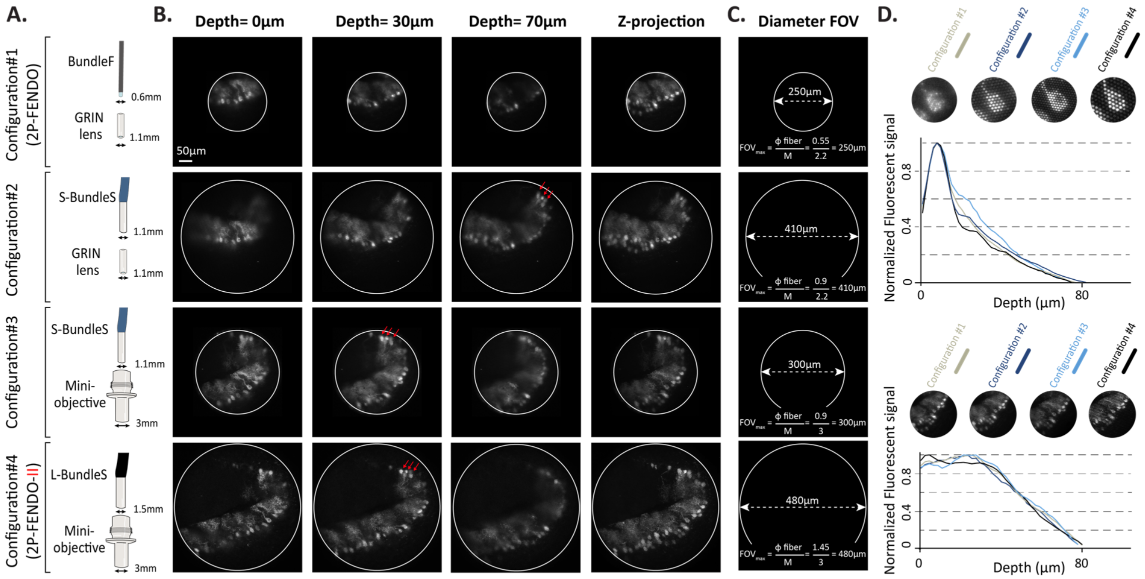

Figure S1: Imaging capability comparison between multiple configurations of the 2P-FENDO, related to Table 2.

(A). Schematic representation of the fibers and lenses for configuration#1 (BunfleF + GRIN lens), configuration#2 (S-BundleS + GRIN lens), configuration#3 (S-BundleS + min-objective), and configuration#4 (L-BundleS + mini-objective), with the corresponding external diameter value.

(B). Imaging on the same GCaMP6-positive 100 µm cerebellar slice with all proposed configurations of the system at multiple depth from the section's surface (0, 30 and 70µm), with z-projection. Red arrows point group of cells in periphery of the FOVs focus at 70µm for configuration#2, and 30µm for configurations #3 and #4, to illustrate the field curvature effect of the GRIN lens.

(C). Calculated diameter of each FOV. Configuration#4 is kept for all subsequent experiment and named 2P-FENDO-II.

(D). Normalized fluorescence signal relative to the depth of imaging tested independently for each configuration on the cerebellar slices of 80 µm. Signals were taken for a small ROI over a single Purkinje Cell (upper), or as an average of a 100µm ROI centred in the FOV (lower).

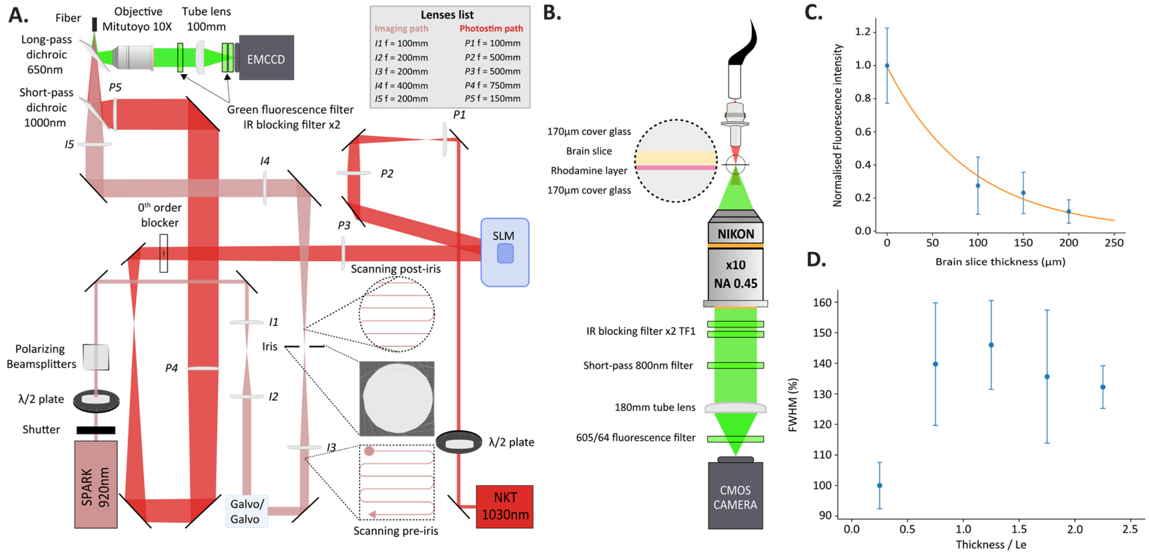

Figure S2: Description of the setup and the evolution of axial resolution with scattering tissue, related to Figure 1 and STAR★METHODS setup description.

(A). Complete schematic of the 2P-FENDO-II setup.

(B). Schematic of the transmissive configuration used to measure the evolution of the axial confinement of 2P photostimulation spot as a function of the brain slices thickness.

(C). Evolution of the fluorescence intensity signal with increasing brain slice thickness. Intensity values were averaged over multiple spot positions within the same FOV and across multiple FOVs. The orange curve represents an exponential decay fit corresponding to an attenuation length of Le=184µm.

(D). Broadening of the axial resolution as a function of penetration depth. To account for variations in scattering length among different preparations, the x-axis shows the brain slice thickness depth normalized to the corresponding attenuation length (Le). The y-axis represents the percentage increase in axial FWHM relative to the value in the absence of scattering. Based on this data and previously reported attenuation lengths for in vivo brain tissue (typically ~200 µm at 920 nm; Horton et al.[1]), we estimate that at the working depth of 120 µm used in cortical experiments, the axial broadening remains around ~35%.

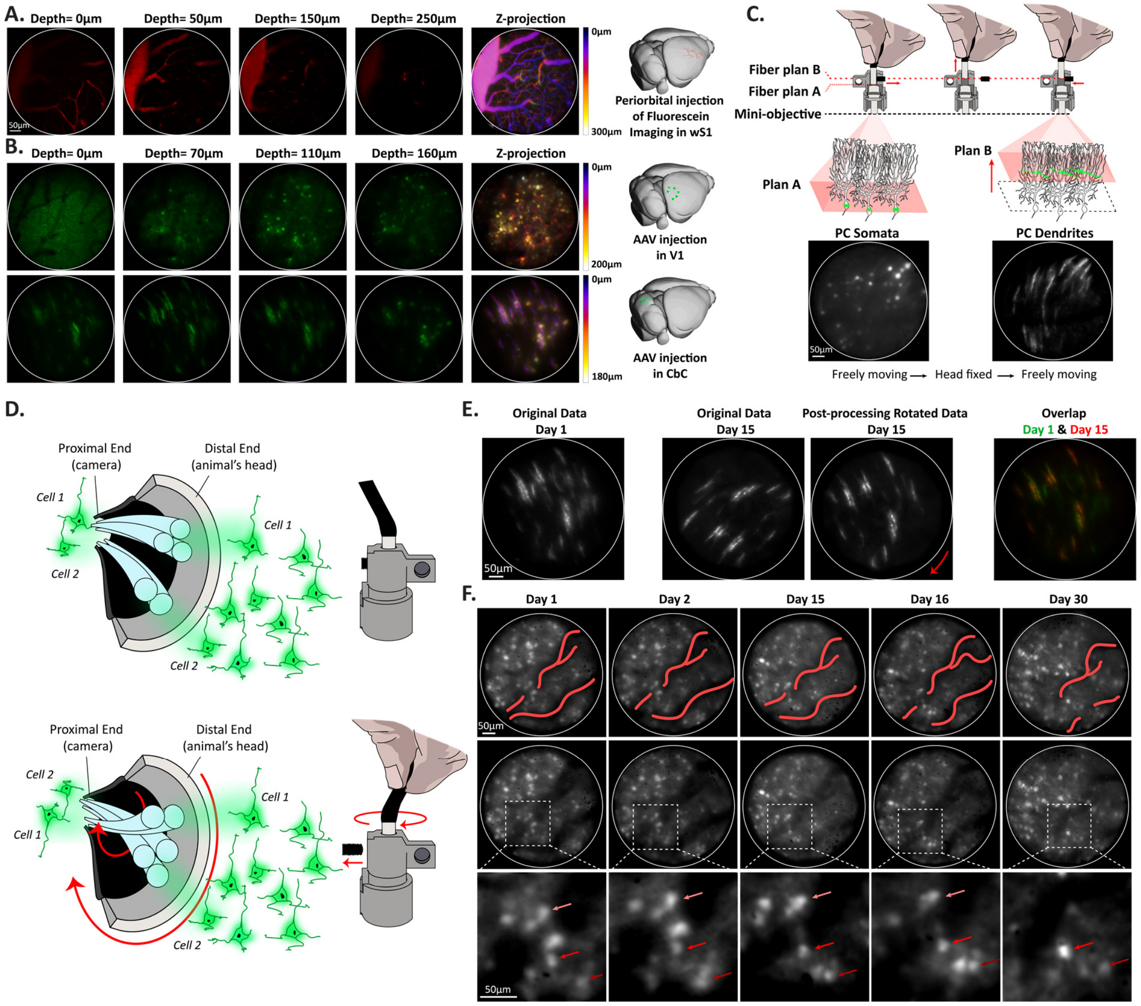

Figure S3: Imaging limits and FOV retrieval technique description, related to Figure 2 and 3.

(A). Z-stack imaging of head fixed mice at 130mW imaging scanning power, periorbitally injected with fluorescein to label blood vessels, with a cranial window over wS1. The images show multiple depths from the surface, and a z-projection of the whole stack color-coded for depth.

(B). Images of jGCaMP7s-labelled neurons in the V1 cortex image at 130mW scanning power (top row) or CbC at 65mW scanning power (bottom row) at multiple depths from the surface, with a z-projection of the whole stack color-coded for depth. Schematic representation of the imaging FOV location for blood vessels or neurons imaging are showed on the right.

(C). Demonstration of the system's flexibility in adjusting the imaging plane depth, with projections of acquisitions at plane A (estimated at 160µm from the surface) and plane B (estimated 50µm<x<90µm from the surface, see also Movie 2).

(D). Schematic illustrating the potential rotational effect of the FOV following manual rotation of the fiber.

(E). Acquisitions of the same FOV and CbC preparation taken 15 days apart, demonstrating the realignment of FOVs after fiber rotation during system reassembly.

(F). Acquisitions of the same FOV and wS1 preparation taken up to 30 days apart, realigned, and compared to track individual neurons across sessions. FOVs are identified based on blood vessels (outlined in red) prior to cells identifications (arrows).

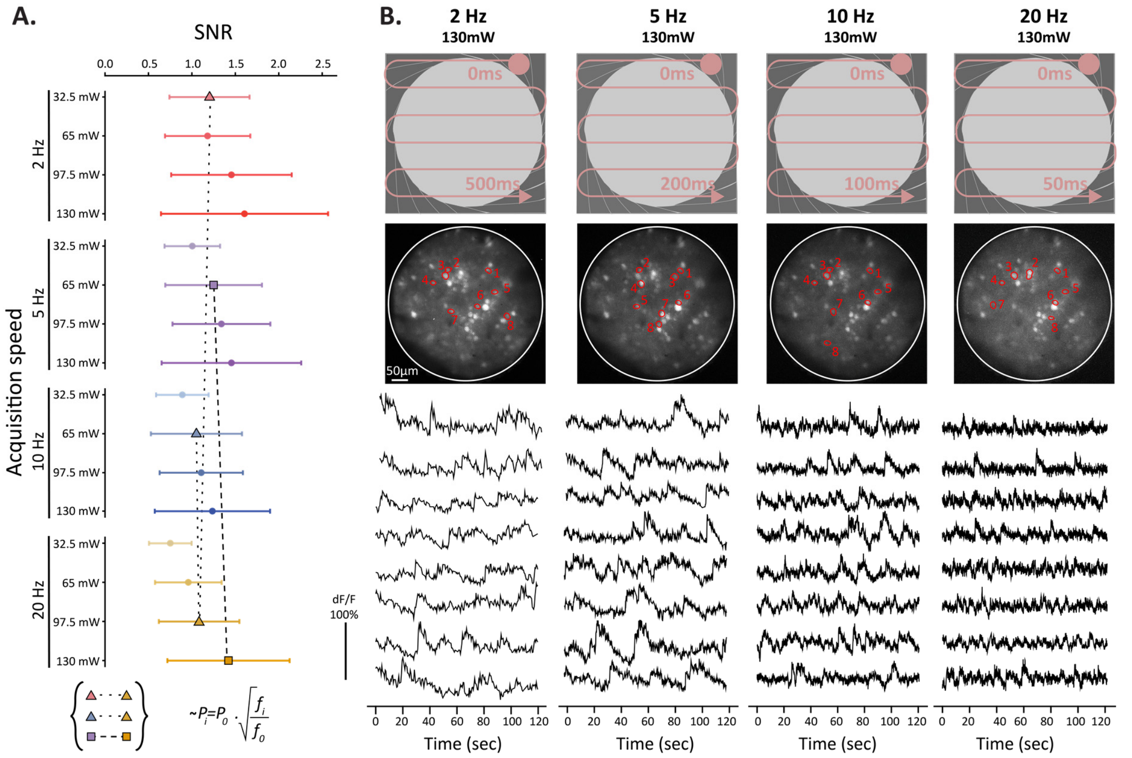

Figure S4: Supplementary data on the maximum achievable imaging speed on a 480µm circular FOV, related to Figure 2.

(A). Imaging acquisitions up to 20Hz with 2P-FENDO-II. Standard deviation of the SNR on a single FOV in wS1 as function of imaging power, 32.5/65/97.5 and 130mW for a scanning spot on the sample plane, and acquisition frame rate for 2Hz (red), 5Hz (purple), 10Hz (blue), and 20Hz (yellow).

(B). Example acquisitions over the same FOV at 2/5/10 and 20Hz for 130mW of laser scanning power. Schemes illustrate the speed of scanning.

Supplemental references

*S*1. Horton, N.G., Wang, K., Kobat, D., Clark, C.G., Wise, F.W., Schaffer, C.B., and Xu, C. (2013). In vivo three-photon microscopy of subcortical structures within an intact mouse brain. Nat Photonics *7*, 205–209. https://doi.org/10.1038/nphoton.2012.336.
